# Application of time lags between light and temperature cycles for growth control based on the circadian clock of *Lactuca sativa* L. seedlings

**DOI:** 10.3389/fpls.2022.994555

**Published:** 2022-10-13

**Authors:** Kosaku Masuda, Tatsuya Yamada, Yuya Kagawa, Hirokazu Fukuda

**Affiliations:** ^1^ Department of Mechanical Engineering, Graduate School of Engineering, Osaka Prefecture University, Sakai, Japan; ^2^ Department of Mechanical Engineering, Graduate School of Engineering, Osaka Metropolitan University, Sakai, Japan

**Keywords:** circadian clock, environmental design, *Lactuca sativa* L., light-dark cycle, phase response curve, plant factory, temperature cycle

## Abstract

The circadian clock plays an important role in agriculture, especially in highly controlled environments, such as plant factories. However, multiple environmental factors have an extremely high degree of freedom, and it is difficult to experimentally search for the optimal design conditions. A recent study demonstrated that the effect of time lags between light and temperature cycles on plant growth could be predicted by the entrainment properties of the circadian clock in *Arabidopsis thaliana*. Based on this prediction, it was possible to control plant growth by adjusting the time lag. However, for application in plant factories, it is necessary to verify the effectiveness of this method using commercial vegetables, such as leaf lettuce. In this study, we investigated the entrainment properties of the circadian clock and the effect of the time lag between light and temperature cycles on circadian rhythms and plant growth in *Lactuca sativa* L. seedlings. For evaluation of circadian rhythms, we used transgenic *L. sativa* L. with a luciferase reporter in the experiment and a phase oscillator model in the simulation. We found that the entrainment properties for the light and temperature stimuli and the effects of time lags on circadian rhythm and growth were similar to those of *A. thaliana*. Moreover, we demonstrated that changes in growth under different time lags could be predicted by simulation based on the entrainment properties of the circadian clock. These results showed the importance of designing a cultivation environment that considers the circadian clock and demonstrated a series of methods to achieve this.

## Introduction

Leaf lettuce (*Lactuca sativa* L.) is one of the major products of plant factories, and extensive research has been conducted on the cultivation of leaf lettuce in artificial environments (Anpo et al., 2018; Kozai et al., 2019). Lighting is one of the most important factors influencing growth in cultivation environments, and lighting intensity, light wavelength, and photoperiod influence the yield and quality of lettuce (Kang et al., 2013; Son et al., 2016). Temperature is another critical factor, and the combination of light and temperature greatly influence lettuce productivity (Carotti et al., 2021; Zhou et al., 2022). Therefore, optimizing light and temperature is essential during lettuce cultivation. Conversely, in plant factories, lettuce is often cultivated under a light–dark cycle, which causes temperature fluctuation. Therefore, the combination of light and temperature requires dynamic design. However, it is difficult to evaluate growth in a changing environment because of the large number of combinations of environmental factors that may vary over time. Therefore, the cultivation environment design should be rooted in the Speaking Plant Approach (SPA), which optimizes the dynamic environment based on the real-time measurement of plant phenotypes (Hashimoto, 1989; Katsoulas et al., 2016).

The circadian clock is a physiological system that adapts to diurnal environmental changes, such as light and temperature, over a 24-h period. It is closely related to various plant activities such as growth, flowering, and stress responses (Dodd et al., 2014; Creux and Harmer, 2019). In *Arabidopsis thaliana* and *Lactuca sativa* L, growth is maximized when the period of the cycle is close to the free-running period of the plant’s circadian clock, verifying the relationship between the period of the light-dark cycle and plant growth (Dodd et al., 2005; Higashi et al., 2015). In addition, the circadian clock is reportedly an important factor influencing the growth of lettuce seedlings in plant factories (Moriyuki and Fukuda, 2016; Nagano et al., 2019). Consequently, the design of a cultivation environment based on the circadian clock may improve productivity in plant factories.

Light and temperature influence the plant circadian clock, and their combination further impacts circadian rhythms (Gil and Park, 2019; Wang et al., 2020). Moreover, according to a recent study, the difference in timing (time lag) between the light and temperature cycles changes plant growth and circadian rhythms in *A. thaliana*, and there are some correlations between plant growth and circadian rhythms (Masuda et al., 2021b). Masuda et al. (2021b) also proposed a method for predicting the effect of time lags on plant growth by predicting circadian rhythms based on a simulation using the phase response curve (PRC), which is a summary of response to the stimuli at each phase of the circadian rhythm and represents the entrainment properties with environmental cues (Johnson et al., 2003). Using the method, it is possible to design time lags among environmental cues based on PRCs to control plant growth. Moreover, Masuda et al. (2021a) proposed a simple PRC estimation method. In the method, the amplitude and phase responses of circadian rhythms to the stimuli at the extremely low amplitude state, which are termed “singularity response (SR)” and reflect the primal characteristics of PRCs, are measured. The method can estimate the PRC in a single measurement, whereas conventional methods require four or more experiments to measure the responses at each phase of the circadian rhythm. By combining the time lag design method based on PRC and the PRC estimation method, we can design a cultivation environment by considering the circadian clock through a few experiments and simulations. Since controlling time lags has a relatively low cost but has a large effect on plant growth, the optimization of time lag is important in the environmental design of plant factories. However, these methods have only been demonstrated for *A. thaliana*, and there has been no verification that these methods can be applied to crop species.

In this study, we verified a series of methods for designing a cultivation environment based on the circadian clock using seedlings of leaf lettuce. First, we verified the PRC estimation method by measuring SR to the light and temperature stimuli. We measured SRs to dark and temperature stimuli and compared the results with those of A. *thaliana* measured in a previous study (Masuda et al., 2021a). Second, we measured the effects of the time lag between the light and temperature cycles on circadian rhythms and growth in leaf lettuce to evaluate the relationship between growth and rhythm in these conditions. Finally, we performed a simulation based on PRCs to predict the circadian rhythm under the time-lag conditions and compared the results of the experiments and the simulation. We then predicted plant growth at different time lags using the relationship between growth and circadian rhythm in the experiments and the simulation to validate the time-lag design method based on PRCs.

## Materials and method

### Model for predicting plant growth for environmental time-lags

The following is a summary of the sequence of steps applied in the construction of the growth prediction model for environmental time-lags (**Figure S1**).

1)Measurement of SR

SR is measured for two or more stimuli for which we want to design a time lag, and PRC is estimated according to the method of a previous study (Masuda et al., 2021a). If necessary, the free-running period is also obtained at the same time. In the present study, we estimated PRC for light and temperature stimuli.

2) Evaluation of growth and circadian rhythms

To confirm the changes in growth and circadian rhythms under time-lag environments, the degrees of growth (such as weight and plant size) and circadian rhythm parameters (amplitude and phase) in the environments are measured. Subsequently, the relationship between growth and the circadian rhythm is determined. In the present study, growth and circadian rhythm in leaf lettuce under the light and temperature cycles were measured with time lags at 6-hour intervals.

3) Simulation to predict circadian rhythms

Based on the simulation using PRC obtained in 1), change in the parameters of the circadian rhythm in response to change in time lag is obtained. By comparing the result with the parameters of the circadian rhythm obtained in 2), the value estimated based on the simulation is corrected.

4) Construction of growth prediction model for environmental time-lags

By integrating the relationship between circadian rhythm and growth obtained in 2) and the model for predicting changes in circadian rhythm in response to time lags obtained in 3), we can construct a model for predicting growth in response to time lags from PRC, which allows simulations to predict changes in growth due to time lags even under conditions other than those obtained experimentally. Using the model, we can obtain the optimal time lag for plant growth.

### Growth conditions

To investigate the circadian rhythm in leaf lettuce, we used seedlings of transgenic *L. sativa* L. [cv. Green Wave (GW) and Cos] *AtCCA1*::*LUC*, which carries luciferase reporters driven by promoters of the *A. thaliana* clock gene *CCA1* (Ukai et al., 2012; Higashi et al., 2014). Plants were grown on gellan gum-solidified Murashige-Skoog medium (M-S Plant Salt Mixture, Wako Chemical Co., Tokyo, Japan) of the standard concentration with 2% (w/v) sucrose in 40-mm-diameter dishes under L/D = 12/12 h and 100 μmol m^−2^ s^−1^ fluorescent white light in a temperature-controlled chamber (MIR-351H, SANYO Electric Co., Ltd., Osaka, Japan) at 22 ± 0.5 °C for 7 days. The plants were treated with 500 μL of 1 mM luciferin, D-Luciferin Firefly, potassium salt (BIOSYNTH AG, Staad Switzerland) in water, 24 h before the start of bioluminescence monitoring. The dishes were sealed to prevent gaseous exchange with the atmosphere outside. Bioluminescence measurements were carried out using an automatic luminescence measuring system known as *Kondotron*, which detects the luminescence of the entire plant by a photomultiplier tube (Hamamatsu H7360-01MOD, Hamamatsu Photonics KK, Shizuoka, Japan) enclosed in a light-tight box (**Figure S2**; Kondo et al., 1993; Fukuda et al., 2013). Each plant was on a turntable, which was located under the PMT and rotated sequentially every 20 min under the control of a computer. We measured bioluminescence under light-emitting diode (LED) illumination with a red LED (60 μmol m^−2^ s^−1^ under light conditions and 0 μmol m^−2^ s^−1^ under dark conditions, λ_p_ = 660 nm) and blue LED (15 μmol m^−2^ s^−1^ under light conditions and 0 μmol m^−2^ s^−1^ under dark conditions, λ_p_ = 470 nm) in a growth chamber (MIR-553, SANYO Electric Co., Ltd., Osaka, Japan) with temperature controlled at 22 ± 0.5°C.

### SR measurement

We measured the SR to 8-h darkness, 4-h 32°C (+10°C), or 4-h 12°C (−10°C) stimuli, as measured in a previous study with *A. thaliana* (Masuda et al., 2021a). The single stimuli do not severely impair lettuce growth, but induce a clear response in circadian rhythms. 8-h darkness, 4-h +10°C, or 4-h −10°C stimuli were applied 262 h, 264 h, and 264 h after the beginning of measurement, respectively. LED illumination was controlled by a computer and the temperature stimuli were applied by changing the set temperature of the growth chamber. Each monitoring was carried out for 14 days using 20 individuals for GW and Cos once. Individuals that did not exhibit bioluminescence were excluded from the calculation.

We obtained the SR parameters using the following calculation (Masuda et al., 2021a).

1) Normalization of bioluminescence: The bioluminescence was normalized as


(1) $${\bar{l}}_{i}=\frac{1}{2w+1}\sum_{k=-w}^{w}l_{i+k},$$

(2) $$\;L_{i}=\left(l_{i}-{\bar{l}}_{i}\right)/{\bar{l}}_{i}$$

where *l*_*i*_ is the *i*th time point of bioluminescence, ${\bar{l}}_{i}$ is the moving averaged bioluminescence, and *L*_*i*_ is the normalized bioluminescence*. w* is the half-window size of the moving average. The measurement intervals were 20 min; therefore, *w* was set to 36 for 24-h window averaging.

2) Peak and trough detection: For normalized bioluminescence signal {*L*_*k*_:_*k*_=1, 2, … } , the slope *s*_*k*_ at the *k*th data point was calculated as


(3) $$s_{k}=\frac{1}{w}\sum_{j=1}^{\text{w}}\left(L_{k+j}-L_{k-j}\right).$$

The moving average window was set as *w* = 24. Peak point *l* of the bioluminescence oscillation is defined as the point at which *s*_*l*_≥0 and *s*_*l*+1_<0 , whereas trough point *m* is defined as the point at which *s*_*m*_≤0 and *s*_*m*+1_>0 .

3) Definition of SR: Denoting the stimulus end time by *t*_e_ , the first peak time of the bioluminescence signal after the end of stimulus by *t*_p_ , and the natural period by *τ*_0_ , the reset phase *Θ* ^′^ is defined as:


(4) $$\Theta^{\prime }=-\frac{t_{\text{p}}-t_{\text{e}}}{\tau_{0}}\times 2\pi .$$

The free-running period *τ*_0_ was taken as the average for the first several days under constant light. As the first peak of *AtCCA1*::*LUC* appeared approximately 2 h after the start of the measurement (Higashi et al., 2014), Θ' was transformed to circadian time (CT) as CT=Θ'/2*π*×24+2 mod 24.

The reset amplitude was defined as


(5) $$R^{\prime }=\frac{\max\left\{L_{k}\right\}-\min\left\{L_{k}\right\}}{2},\;\;k/3\;>t_{\text{e}}+12.$$

The parameters of the phase-sensitive function *Z*(*θ*)=*a*sin(*θ*−*α*) , which represents the change in frequency under stimulation, were calculated according to a previous study (Masuda et al., 2021a). The correction parameter for *R* ^′^ shown in a previous study (*β* = 1.61) was also used in the present study.

We used the Tukey-Kramer test for multiple comparisons of period and amplitude at a 0.05 significance level. We also used the Watson-Williams test with Bonferroni correction for multiple comparisons of Θ' at a 0.05 significance level (Watson and Williams, 1956). We performed the statistical tests using R (version 4.1.1; https://www.r-project.org/). We used the “multcomp” package in R for the Tukey-Kramer test and the “circular” package for the Watson-Williams test.

### Time lag experiments

The photoperiod was set to L/D = 12/12 h. Periodic temperature stimuli (+10°C or −10°C from 22°C for 12 h within a 24 h period) were applied with four time-lags (*Δt* = 0, 6, 12, and 18 h after turning on the light). As a control, experiments with L/D = 12/12 h at 22°C without temperature cycles were also performed. Each experiment was carried out for seven days using six individuals for GW and seven for cos once. After measuring bioluminescence, we measured both the fresh weight of the aerial part and the projected leaf area (PLA) to evaluate the effects on productivity for commercial agriculture (Kozai et al., 2019).

The phase and amplitude were calculated according to Masuda et al. (2021b). Bioluminescence of *AtCCA1*::*LUC* was initially normalized in the same manner as in the SR experiment (Eqs. (1) and (2)). To determine the amplitude and phase of the *AtCCA1* rhythm, we obtained the first Fourier series, *A*cos*θ*(*t*) , of *L*_*i*_ . The first Fourier component is obtained using the following equation:


$$a_{1}=\frac{2}{h-2w}\sum_{i=1+w}^{h-w}L_{i}\cos\left(2\pi \frac{i\Delta s}{T}\right),\;\;\;$$

(6) $$b_{1}=\frac{2}{h-2w}\sum_{i=1+w}^{h-w}L_{i}\sin\left(2\pi \frac{i\Delta s}{T}\right),\;\;\;$$

where *T* = 24 h and the measurement interval Δ*s* = 1/3 h. *h* is the number of time-course data points (*h* = 168 × 3). *L*_*i*_ is the normalized bioluminescence, and *w* is the moving average window and *w* = 24. Using *a*_1_ and *b*_1_ , amplitudes *A* and phase *θ*(*t*) were determined as follows:


(7) $$A=\sqrt{a_{1}^{2}+b_{1}^{2}},$$

(8) $$\theta \left(t\right)=2\pi \frac{t}{T}-\theta_{1},$$

(9) $$\theta_{1}=\tan^{-1}\frac{b_{1}}{a_{1}}.$$

where *θ*_1_ indicates the phase delay in the *AtCCA1* rhythm in response to the light-dark cycles, i.e., the locking phase, which appears at light-on (*θ*(0)=−*θ*_1_ ). To distinguish them from the values obtained in simulation, *A* and *θ*_1_ are termed experimental amplitude and phase, respectively. We used the Tukey-Kramer test for multiple comparisons of *A* at a significance level of 0.05. We also used the Watson-Williams test with Bonferroni correction for multiple comparisons of *θ*_1_ at a significance level of 0.05 (Watson and Williams, 1956). The mean of the locking phase ${\bar{\theta }}_{1}$ (which is termed the circular mean) is defined as $\arg\left\{\frac{1}{N}\sum_{j=1}^{N}e^{\text{i}\theta_{1,\;\;j}}\right\}$ , where *θ*_1,*j*_ is the locking phase (rad) of the *j*th individual. We used the “multcomp” package in R for the Tukey-Kramer test and the “circular” package for the Watson-Williams test.

### Numerical simulation of circadian rhythm

Since the plant circadian clock has a very large number of cellular oscillators, individual-level circadian rhythms represent synchronization states among the oscillators (Masuda et al., 2021b). Under light and temperature cycles, each oscillator was modulated through phase responses for each stimulation. Therefore, the population dynamics of cellular oscillators and their synchronization states are described as follows:


(10) $$\frac{d\phi_{j}}{dt}=\omega_{j}+p_{\text{D}}\left(t\right)Z_{\text{D}}\left(\phi_{j}\right)+p_{\text{T}}\left(t\right)Z_{\text{T}}\left(\phi_{j}\right)+\frac{K}{N}\sum_{k=1}^{N}\text{sin}\left(\phi_{k}-\phi_{j}\right),$$

(11) $$R\left(t\right)e^{\text{iΦ}\left(t\right)}=\frac{1}{N}\sum_{j=1}^{N}e^{\text{i}\phi_{j}\left(t\right)}\;,$$

where *ϕ*_*j*_ and *ω*_*j*_ are the phase and natural frequencies of the *j*th oscillator, respectively. *K* represents the coupling strength and *N* is the number of oscillators. *ω*_*j*_ has a normal distribution with standard deviation σ_ω_ and mean value ω_0_. *p*_D_(*t*) and *p*_T_(*t*) indicate the presence of dark stimulus and +10°C or −10°C stimuli, respectively (*p*(*t*)=1 for stimulus-on, *p*(*t*)=0 for stimulus-off). These are described as follows:


(12) $$p_{\text{D}}\left(t\right)=\{\begin{array}{c} 1,\;\;12+24m\leq t<24+24m\\ 0,\;\;other\;\;\;\;\;\;\;\;\;\;\;\;\;\;\;\;\;\; \end{array}$$

(13) $$p_{\text{T}}\left(t\right)=\{\begin{array}{c} 1,\;\;\Delta t+24m\leq t<\Delta t+12+24m\\ 0,\;\;other\;\;\;\;\;\;\;\;\;\;\;\;\;\;\;\;\;\;\;\;\; \end{array}$$

where *m* is an integer. *Z*_D_(*ϕ*) and *Z*_T_(*ϕ*) are the phase-sensitivity functions for the dark and temperature stimuli, respectively. These are described using the following formula: *Z*(*ϕ*)=*a*sin(*ϕ*−*α*) . We used the values of *a* and *α* for 8 h darkness and 4 h ±10°C stimuli obtained in the SR experiments. In addition, the collective rhythm *X* of the oscillators is denoted by

X(t)=1N∑j=1Ncos(ϕj(t))=R(t)cos(Φ(t))

, *R*(*t*) and Φ(*t*) correspond to the amplitude and phase of the individual-level rhythms, respectively. In this study, the parameters were set to *N* = 1000, *K* = 0.01, *ω* = 2π/*τ*_0_ h^-1^ rad, and σ_ω_ = 0.2 ω_0_ as in a previous study (Masuda et al., 2021b). *Δ t* varied from 0 to 24 in 0.24 increments. Calculations were performed using the Runge-Kutta method with a time increment of 0.01 h up to *t* = 168 h. *R*(*t*) and Φ(*t*) were averaged over the entire simulation period, and averaged *R*(*t*) and Φ(*t*) are termed computational amplitude and phase, respectively, to distinguish them from those obtained in the experiments. The program was coded in C++ using Visual Studio 2019 (Version 16.11.18, Microsoft Corporation, Redmond, WA, US).

## Results

### SR measurement

**Figure 1:**
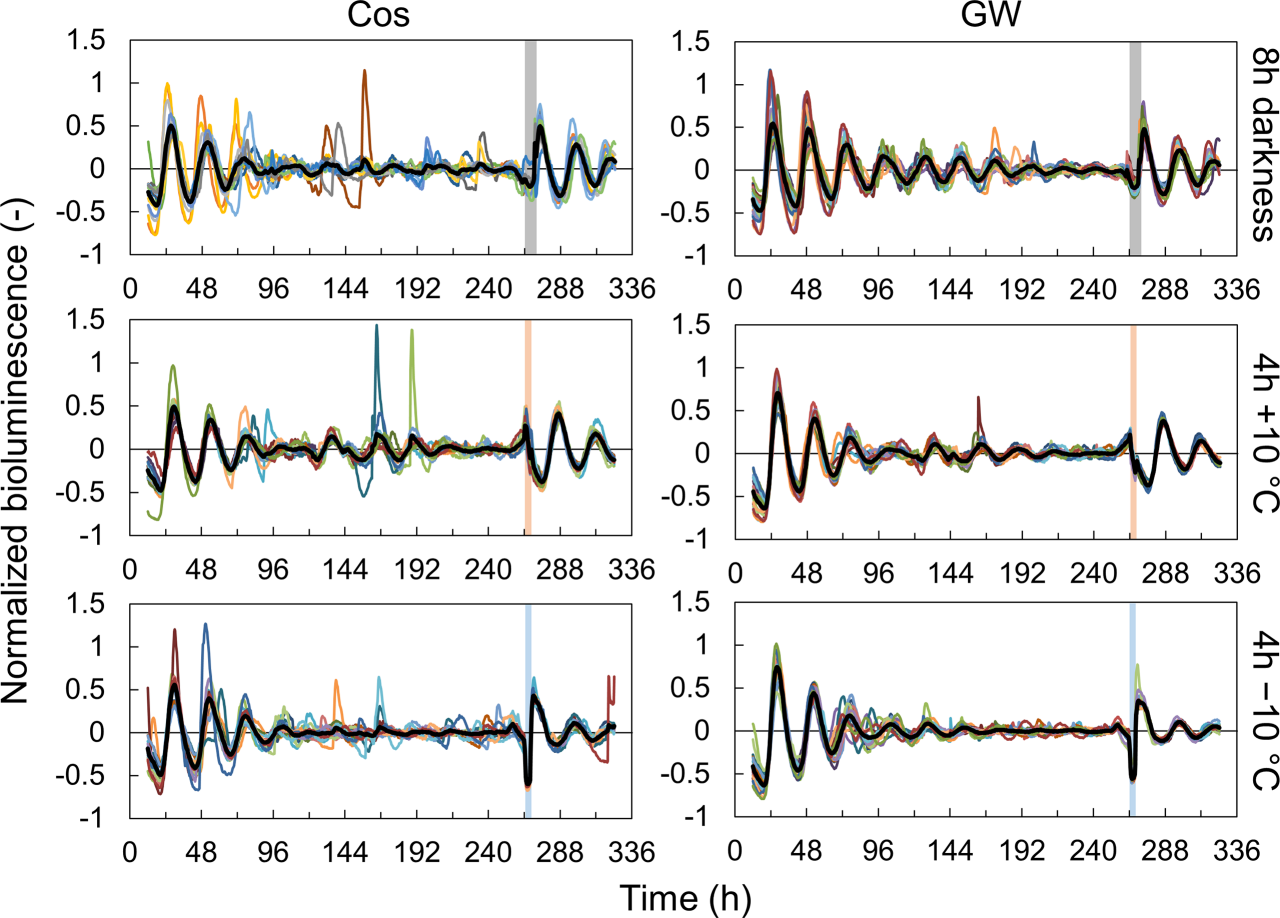
Singularity responses to 8-h darkness, 4-h +10°C, and 4-h −10°C stimuli in *Lactuca sativa* L. (cv. Cos and Green Wave) during an experiment to investigate the effect of time lags in light: dark and temperature cycles during cultivation. Colored lines represent individual bioluminescence, and black lines represent mean normalized bioluminescence (*n* = 13 in 8-h darkness, 15 in 4-h +10°C, and 16 in 4-h −10°C in Cos, and *n* = 20 in all conditions in GreenWave). Vertical bars represent the stimulation.

**Figure 2:**
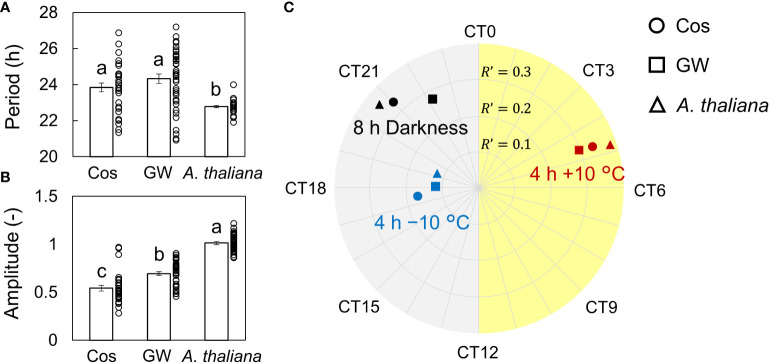
Periods, amplitude, and singularity response (SR) to dark and temperature stimuli of *Lactuca sativa* L. during an experiment to investigate the effect of time lags in light: dark and temperature cycles during cultivation. **(A)** Natural periods of leaf lettuce (cv. Cos and GW), and *Arabidopsis thaliana* (mean ± SEM; *n* = 31 individuals in Cos and *A thaliana*, and 40 in GW). **(B)** Amplitude of Cos and GW and *A thaliana* (mean ± SEM; *n* = 31 in Cos and *A thaliana*, and 40 in GW). The circles indicate the individual data points. Two values which have no common letter show significant differences (Tukey-Kramer test, *p*< 0.05). **(C)** SR to 8-h darkness and 4 h of ±10°C stimuli of Cos and GW and *A thaliana*. *R’* is described as the distance from the center point. CT is circadian time, and CT2 is defined as the peak time of the bioluminescence of *AtCCA1*::*LUC*.

First, we measured the singularity responses using *L. sativa* L. *AtCCA1*::*LUC* (cv. Cos and GW) in response to light and temperature stimuli (**Figures 1**, **S3**). The amplitude of the rhythm decreased over time and became almost zero (singularity state) on day 11 in both the Cos and GW. After the light and temperature stimulation, the rhythm of each individual was reset to the same phase and amplitude. This result shows that SR can also be measured in the circadian rhythm of leaf lettuce. SRs were observed for both light and temperature stimuli, and the reset phase and amplitude depended on the stimulus (**Figure S3**). The period, natural amplitude, and SR parameters in leaf lettuce and *A. thaliana* were compared (**Figure 2**). The periods in leaf lettuce were longer than those in *A. thaliana*, and there was no difference between GW and Cos (**Figure 2A**; **Table S1**). The natural amplitude, (the amplitude at the beginning of the measurement) of leaf lettuce was smaller than that of *A. thaliana*, and that of Cos was smaller than that of GW (**Figure 2B**; **Table S1**). The SR phase of the leaf lettuce was similar to that of *A. thaliana* (**Figure 2C**; **Tables S2**, **S3**). The SR to dark stimulus showed the phase later at night, and +10°C and −10°C stimuli showed the phases at midday and midnight, respectively. The SR amplitude of leaf lettuce was similar to that of *A. thaliana*. The dark and +10°C stimuli showed similar amplitudes, and −10°C showed smaller amplitudes than +10°C. These results indicate that SR can be used to assess the entrainment properties of leaf lettuce, as well as *A. thaliana*. The SR amplitude of Cos was larger than that of GW (Welch’s *t*-test, *p*< 0.05, in each of dark and ±10°C stimuli), although the natural amplitude of Cos was smaller than that of GW. The phase of SRs to 8-h darkness was also significantly different between Cos and GW (Watson-Williams test, *p*< 0.05).

### Effects of time lags on growth and circadian rhythms

**Figure 3:**
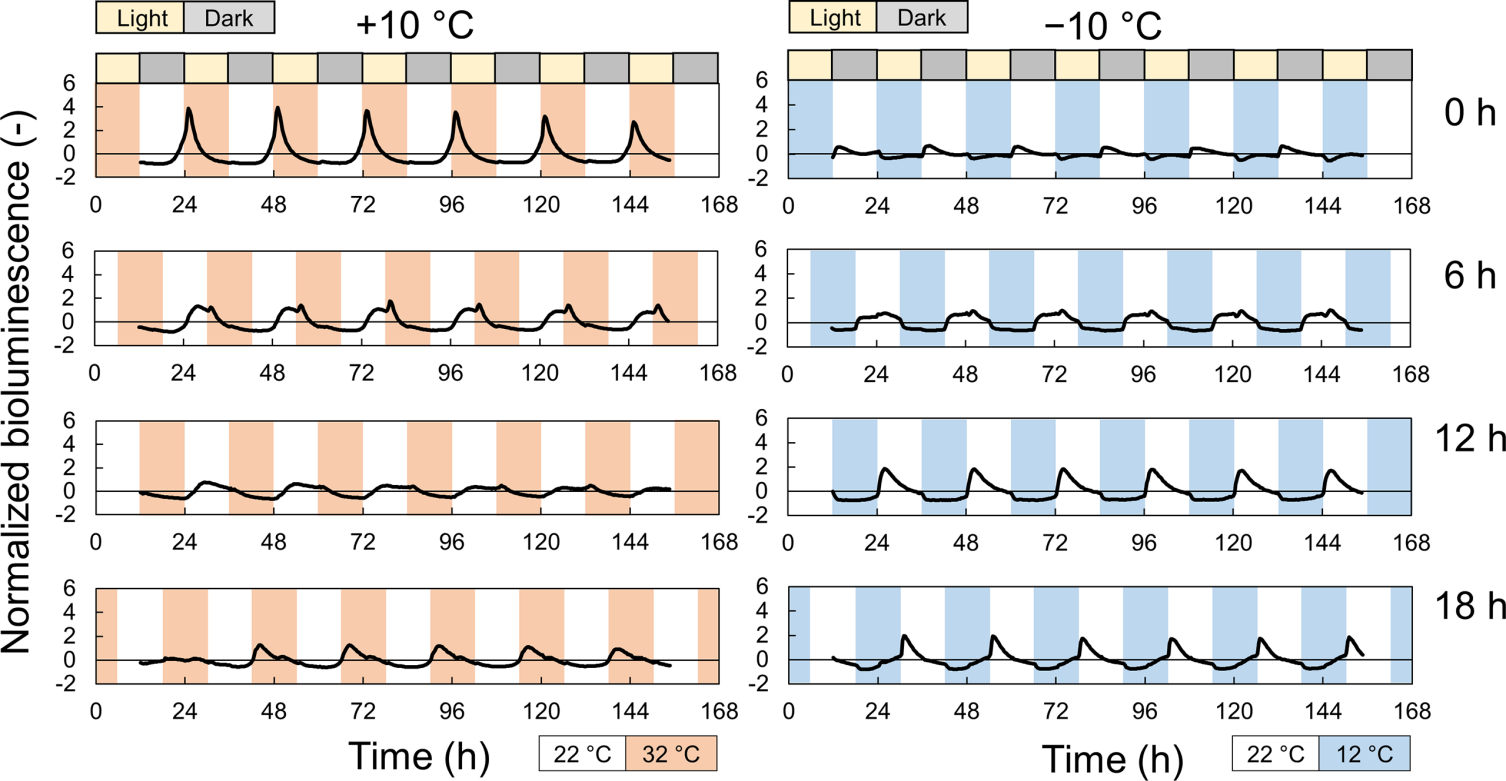
Normalized bioluminescence of *AtCCA1*::*LUC* in *Lactuca sativa* L. ‘Cos’ under the light and ±10°C temperature cycles with different time lags during an experiment to investigate the effect of time lags in light: dark and temperature cycles during cultivation. Photoperiod is L/D = 12/12 h. Periodic temperature stimuli (+10°C [orange] or −10°C [blue] from 22°C for 12 h within a 24-h period) were applied with four time-lags (*Δt* = 0, 6, 12, and 18 h after turning on the light). Each data is the average of 7 individuals.

**Figure 4:**
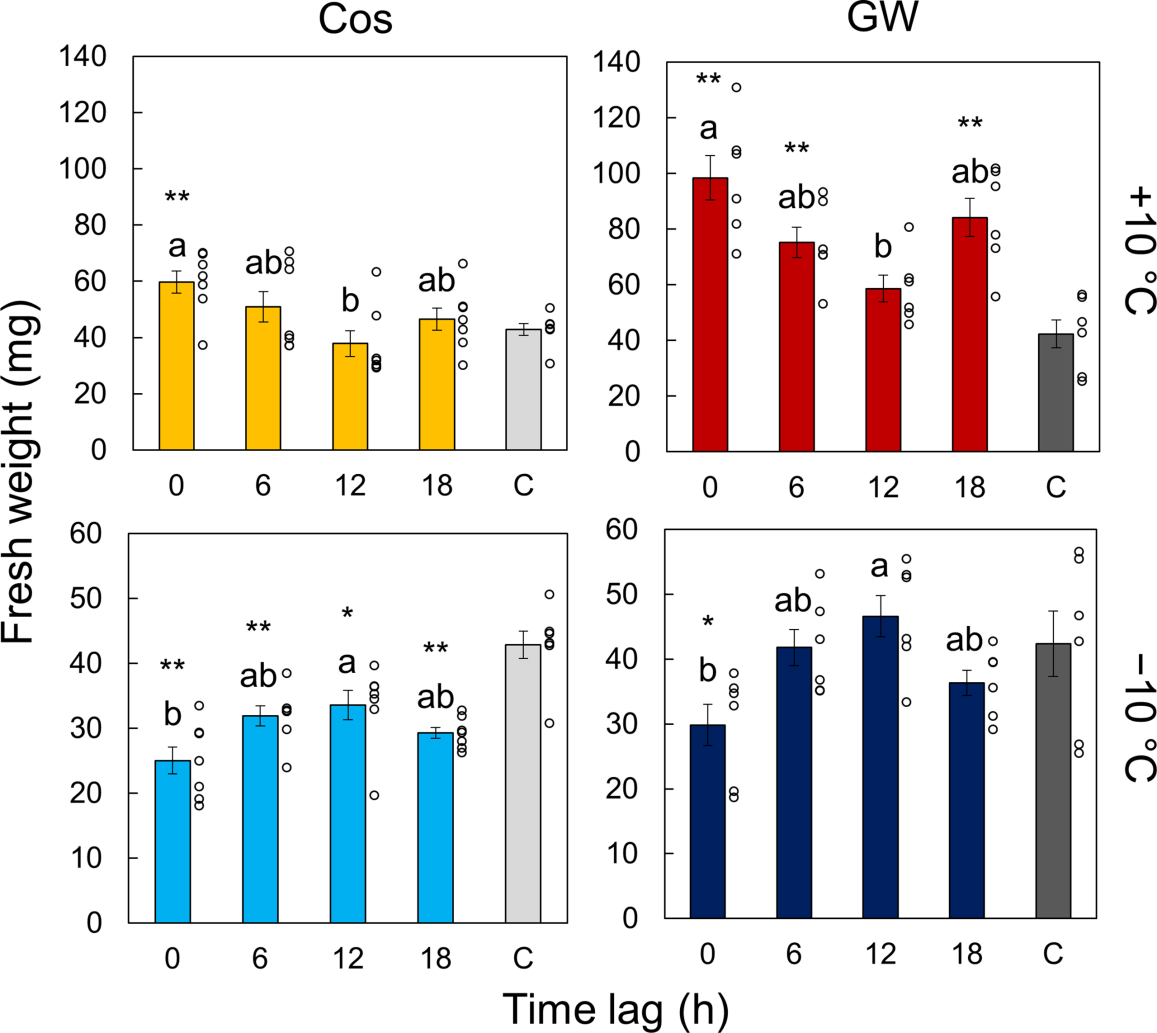
Effect of the time lag on plant growth. The control condition is labeled C. Each data point is the mean of *n* = 7 individuals in leaf lettuce (*Lactuca sativa* L.) ‘Cos’ and 6 in ‘GW’. Error bars indicate standard error. The circles indicate the individual data points. Two conditions that do not have the same letter indicate significant differences for each panel (Tukey-Kramer test, *p*< 0.05). Asterisks indicate significance differences with the control condition (Welch’s *t*-test, *p< 0.05, **p< 0.01).

We measured the effect of time lags between light and temperature cycles on the growth of the plant and circadian rhythms. **Figures 3** and **S4** show the normalized bioluminescence of Cos and GW under light/dark and hot/cold cycles with different time lags. The circadian rhythms were modulated by the time lag, and the amplitude was especially suppressed at Δ*t* =12 at +10°C and at Δ*t* =0 at −10°C (**Figure S5**). The amplitude at Δ*t* =18 h at +10°C was also suppressed in Cos but not in GW. The peak time also varied depending on the time lags, but the difference between the Cos and GW in the peak time was smaller than that in the amplitude (**Figure S6**). **Figure 4** shows the fresh weight at each time lag. At +10°C, the fresh weight was the highest at Δ*t* = 0 h and the lowest at Δ*t* = 12 h for both Cos and GW. The lowest fresh weights were 37% and 40% smaller than the highest fresh weights in the Cos and GW, respectively. Conversely, the fresh weight was the highest at Δ*t* = 12 h and the lowest at Δ*t* = 0 h for both Cos and GW at−10°C. The lowest fresh weights were 25% and 36% smaller than the highest ones in Cos and GW at −10°C, respectively. Compared with the control condition, the fresh weight at +10°C was 39% and 132% higher at the maximum in Cos and GW, respectively. However, the fresh weight at −10°C was 22% lower than that of the control, even at the maximum in Cos. The PLA showed similar changes in response to the time lag (**Figure S7**), indicating that the changes in fresh weight were not simply changes in water content, but changes in growth rate.

**Figure 5:**
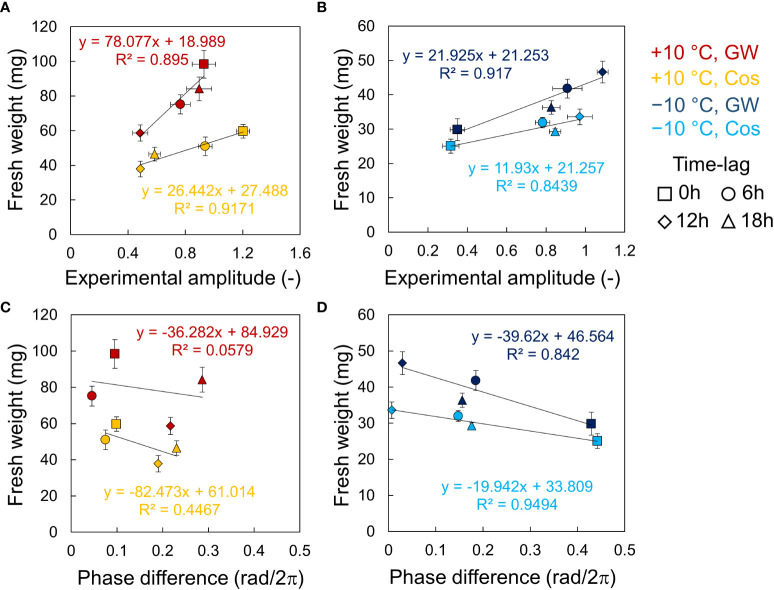
Relationship between fresh weights of leaf lettuce (*Lactuca sativa* L.) and *AtCCA1* rhythms in the conditions of L/D = 12/12 h and 12 h ±10°C cycles. **(A, B)** Relationship between fresh weight and amplitude at +10°C **(A)** and −10°C **(B)**. **(C, D)** Relationship between fresh weight and *Δ θ*_1_ at +10°C **(C)** and −10°C **(D)**. Error bars indicate standard error. The solid lines indicate regression lines. The value of R^2^ indicates the correlation coefficient of determination.

In the relationship between fresh weight and the circadian rhythm, the fresh weight and the amplitude showed high positive correlations at both +10°C and −10°C, for Cos and GW (**Figures 5A, B**). The slopes of the regression lines tended to be larger for larger average weights, but the intercepts were relatively similar. The fresh weight and the phase differences showed negative correlations, as noted in a previous study (Masuda et al., 2021b). However, the correlation coefficients at +10°C were smaller than those at −10°C and so was the amplitude (**Figures 5C, D**).

### Estimation of effects of time lags on circadian rhythms and growth

**Figure 6:**
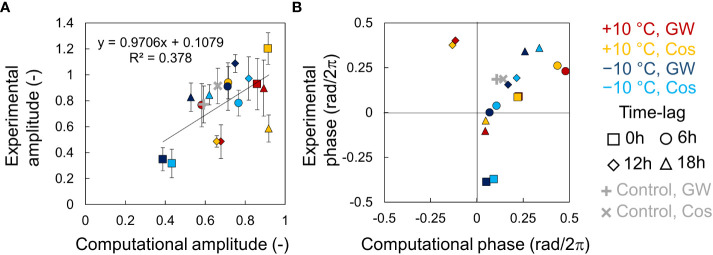
Relationship between the *AtCCA1* rhythms of leaf lettuce (*Lactuca sativa* L.) in the experiments and simulations. **(A)** Correlation between amplitudes in the experiment and simulation (mean ± SD in experiment). Error bars indicate standard deviation. The solid line indicates a regression line. The value of *R* ^2^ indicates the coefficient of determination. **(B)** Correlation between the locking-phases *θ*_1_ in the experiment and the simulation (circular mean in experiment).

We performed simulations using the phase oscillator model based on the entrainment properties of the circadian clock in leaf lettuce (**Figures S8**, **S9**). The amplitude had a positive correlation between the experiments and simulation (**Figure 6A**). However, several points in the heating condition showed different values from the regression line. The relationship between fresh weight and phase was linear, except for a 12-hour time lag at +10°C and a 0-hour time lag at -10°C, where the amplitude was lowest (**Figure 6B**). The low accuracy in these conditions might be due to the difficulty in determining the exact phase of low-amplitude rhythms.

**Figure 7:**
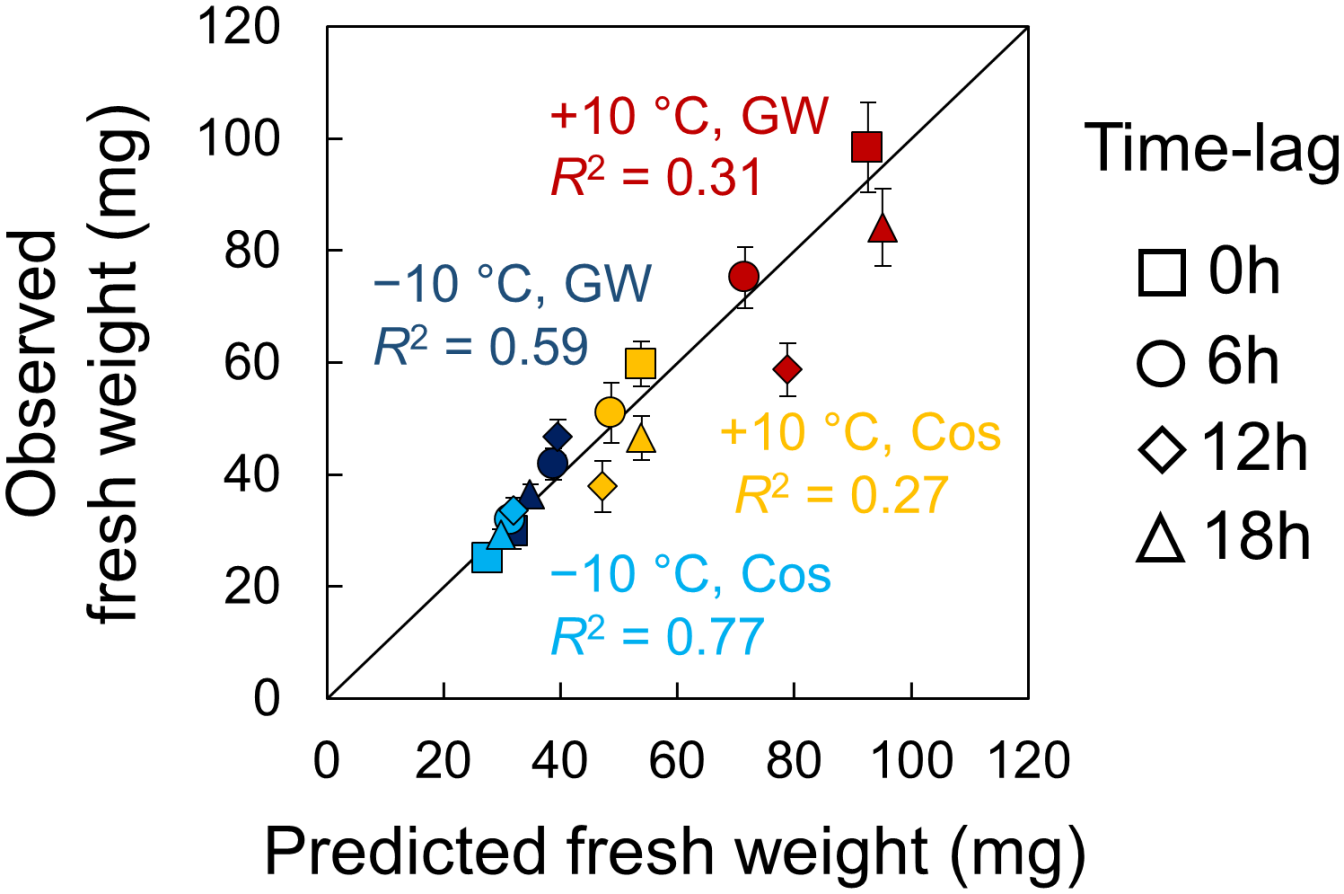
Prediction of fresh weight of leaf lettuce (*Lactuca sativa* L.) by the simulation based on the entrainment properties of circadian clock. Error bars indicate standard error. The value of *R* ^2^ indicates the coefficient of determination for the line with a slope of 1.

There was a relationship between growth and phase in the experiments and simulation, but it was difficult to derive a simple relational equation for it. However, we found positive linear correlations between growth and amplitude in the experiments and simulation. Therefore, we predicted the growth of leaf lettuce seedlings under a time lag using the relationship between growth and amplitude. First, we estimated the experimental amplitude using the equation in **Figure 6A** (Experimental amplitude = 0.9715 × Computational Amplitude + 0.1073), and then, we predicted the fresh weight using the equations in **Figures 5A, B** (e.g., Fresh weight = 78.077 × Experimental amplitude + 18.989 (mg) in +10°C in GW). **Figure 7** shows the predicted fresh weights. The prediction of fresh weight at +10°C showed a moderate accuracy (correlation coefficient *r* = 0.56 in GW, *r* = 0.52 in Cos) because the estimation of amplitude at +10°C was inaccurate at some points. Conversely, the prediction of fresh weight at −10°C showed a high accuracy (correlation coefficient *r* = 0.77 in GW, *r* = 0.88 in Cos). These results indicate that the change in growth, depending on environmental time lags, can be predicted using PRCs. In the simulation, the time lag at which the amplitude is maximum can be obtained at smaller time intervals than in the experiment (**Figure S10**). For both positive and negative temperature conditions, the amplitude was maximal at a time lag that was slightly advanced from the time lag at which growth was maximal in the experiment. This means that optimal environmental time lags can be designed *via* growth prediction based on the proposed method.

## Discussion

We demonstrated a series of measurements and application methods for the circadian clock of leaf lettuce. The results of the SR measurement using leaf lettuce indicate that this simple method can be used to evaluate the relationship between the crop’s circadian clock and environmental stimuli. Time-lag experiments showed a relationship between growth and circadian rhythm under the light and temperature cycles. Furthermore, the simulations using circadian clock characteristics revealed that the circadian rhythm under the light and temperature cycles can be predicted. By integrating these results, we proposed a series of methods to predict the plant growth and design the optimal time lags between multiple environments.

In the present study, we measure SR and changes in growth and circadian rhythm in response to time lags using leaf lettuce seedlings. Conversely, previous studies have used *A. thaliana* to measure SR and the responses of growth and circadian rhythms to time lags in a similar manner (Masuda et al., 2021b). Some differences were found between *Arabidopsis* and leaf lettuce in terms of period, amplitude, and SR to light and temperature stimuli (**Figure 2**; **Table S2**). Differences in period have also been observed within the plant species, highlighting the importance of measuring the differences in entrainment properties (Muranaka et al,. 2015; Greenham et al,. 2017). Under the time-lag conditions, both *A. thaliana* and lettuce showed changes in growth under high temperature conditions; however, no clear changes were observed in *A. thaliana* under low temperature conditions. The average growth was greater under high temperature conditions, while the amount of growth tended to decrease under low temperature conditions (**Figure S11A**). The change has also been observed in other previous studies (Thompson et al., 1998). Therefore, average temperature exists as a parameter independent of time lag and may also alter the effects of time lags on plant growth. In both plants, amplitude and fresh weight had a linear relationship, as did amplitude in the simulation and the experiment. Previous studies have also suggested that circadian rhythms are a key parameter in plant growth assessment in challenging environments (Dakhiya et al., 2017). Therefore, the amplitude of the circadian rhythm may be a common parameter that can describe the relationships among environment, circadian rhythm, and growth.

In previous studies, day-night temperature difference (DIF) has been used to assess the effect of the relationship between light-dark cycles and temperature cycles on plant growth (Myster and Moe, 1995). When the daytime temperature is higher than the nighttime temperature, DIF is positive, and when the daytime temperature is lower than the nighttime temperature, DIF is negative. In the present study, DIF was positive at Δ*t* = 0 h at +10°C and at Δ*t* = 12 h at −10°C, and negative at Δ*t* = 12 h at +10°C and at Δ*t* = 0 h at −10°C. Positive DIF results in greater plant growth than negative DIF in many plant species, including *A*. *thaliana* (Thingnaes et al., 2003), and vegetables such as tomato, eggplant, and cucumber (Patil and Moe, 2009; Inthichack et al., 2013). This is also true in leaf lettuces as demonstrated by the results of this study (**Figure 4**). In addition to the DIF conditions, we also conducted the time-lag experiments in the conditions where the light cycle and temperature cycle were shifted by 6 hours (i.e. Δ*t* = 6 h and 18 h). Thus, time lag is an extension of the concept of DIF. Consequently, plants that show growth changes in response to DIF will also show growth changes in response to time lags. Therefore, our proposed method may be applicable to as many plant species as used in DIF studies.

In this study, we showed that the time lag in light and temperature cycles affects plant growth and circadian rhythms. Several hypotheses can be proposed regarding the mechanism by which the time lag affects growth. Plant morphogenesis depends on light and temperature and these phenomena are termed photomorphogenesis (Wang et al., 2022) and thermomorphogenesis (Delker et al., 2022), respectively. These two processes are mainly controlled by PHYTOCHROME-INTERACTING FACTOR 4 (PIF4) (Zhao and Bao, 2021), and activation of PIF4 is controlled by the circadian clock genes. Therefore, photomorphogenesis and thermomorphogenesis depend on the circadian rhythm (Zhu et al., 2016; Sun et al., 2019). In a time-lag environment, light and temperature cycles change independently, but circadian rhythm can only adopt to one of these cycles. Therefore, the time lag produces a phase difference between either the light or the temperature cycle and the circadian rhythm. This may disturb morphogenesis, resulting in reduced growth. In addition, the photosynthetic efficiency of plants also depends on circadian rhythms (Hennessey and Field, 1991). Therefore, the phase shift and amplitude reduction caused by the time lag may reduce photosynthetic efficiency during the light period.

In the present study, lettuce seedlings were used to evaluate the characteristics of circadian rhythms and the effects of time lags between light and temperature cycles. Therefore, it is difficult to determine whether the same circadian rhythm characteristics and time-lag effects are expressed in mature lettuce from our results. Conversely, since seedling production is one of the most important processes in plant factories, the results of the present study may have some practical utility. We also evaluated the effect of time lags for temperature stimuli of ±10°C in the present study. Since the average fresh weight varied with positive and negative temperature changes and varieties, predictions were performed separately for each condition. However, for practical use, it is necessary to determine the effect of time lag at different average temperatures and different ranges of temperature change, and measuring the effect of time lag individually is time-consuming and costly. Therefore, it is necessary to construct a more comprehensive model that includes such changes. In the present results, the amplitude of the circadian rhythm could be represented based a common model for the two varieties at different average temperatures (**Figure 6**). In contrast, the average fresh weight under time-lag conditions tended to increase with an increase in temperature (**Figure S11A**). In addition, the ranges of change in fresh weight with time lags were proportional to the average fresh weight (**Figure S11B**). Therefore, incorporating such changes into the model may make the model more reliable.

We showed a design method for environmental time lags based on the PRC using leaf lettuce seedlings. Although the control of time lag requires a small cost, it has a large effect on plant growth (**Figure 4**). Hence, controlling the time lag has the potential to improve and adjust plant productivity. However, the temperature in a plant factory is usually controlled by constant target values (Anpo et al., 2018; Kozai et al., 2019). Therefore, it is necessary to evaluate the effects of the presence/absence of temperature cycles on plant growth. However, there is some spatial and temporal unevenness of temperature because of the low capability of air conditioning, different thermal conductivities between air and water, and warming by lighting in the practical cultivation environment. These factors may cause temperature cycles and a time lag with the light cycles. Therefore, our proposed model may also be able to evaluate the effect of spatiotemporal unevenness of the cultivation environment on plant growth.

In the present study, there was a clear correlation between the amplitude of the circadian rhythm in the experiment and plant growth under time-lag conditions. Therefore, to improve the accuracy of growth prediction, it is important to improve the accuracy of amplitude prediction by simulation. One possibility for improving the simulation model is to correctly estimate the entrainment properties of the circadian clock. In a previous study using *A. thaliana*, the parameter *R′* was calibrated using experimentally measured PRC (Masuda et al., 2021a). However, in the present study, we used the same calibration parameters as those obtained in the previous study calibrated for *A. thaliana*. Moreover, the natural amplitude of the circadian rhythm in leaf lettuce differed from that in *A. thaliana*. Therefore, correcting the calibration of the SR parameter may improve accuracy. Nonetheless, although the amplitude of SR at −10°C was smaller than that at +10°C (**Figure 2C**), the change in amplitude depending on time lag at −10°C was larger than that at +10°C in GW (**Figure S3**). These results indicate that the response properties under light/dark and hot/cool cycles differed from the SR parameters, which were measured using shorter stimuli under constant light conditions. Consequently, measuring SRs to the 12-h darkness and temperature stimuli and to the temperature stimuli under darkness may improve the accuracy of the simulation.

Time-lag experiments have shown that the timing of environmental changes has a significant impact on plant growth. Furthermore, it has demonstrated that the optimal environment can be predicted from the characteristics of the circadian clock. This has reiterated the importance of considering the circadian clock during environmental design and also showed the generality and specificity of the relationships between plant growth, environment, and the circadian clock. Further understanding of these relationships and improving the accuracy of simulations will improve plant productivity in plant factories. In this study, we used light/dark and hot/cold cycles to create an environmental time lag. However, there are other factors related to the circadian clock, such as humidity, light wavelength, and sugar signaling (Haydon et al., 2013; Ohara et al., 2015; Mwimba et al., 2018), and other factors in the cultivation environment (e.g., CO2 concentration, nutrient solution, agricultural chemicals) may also be related to the entrainment of circadian rhythms. Furthermore, the plant circadian clock has organ specificity and age dependency (Bordage et al., 2016; Kim et al., 2016). The rhythmicity and entrainment properties also depend on the plant species and varieties (**Figure 2**). These factors may be related to the effects of the time lag. Therefore, by measuring the entrainment properties of these factors using SR, we can design a more detailed cultivation environment.

We measured the circadian rhythm for SR using transgenic leaf lettuce. However, it is difficult to prepare transgenic plants and measurement devices for various crops using this method. Therefore, a more generalized method for the measurement of circadian rhythms is required for general applications. Previous studies have used projected leaf area (PLA) to measure circadian rhythms (Dornbusch et al., 2014; Flood et al., 2016). PLA is also an index of plant growth, and methods for production control in plant factories using image measurements have been proposed (Moriyuki and Fukuda, 2016; Nagano et al., 2019). In the present study, the changes in fresh weight and PLA due to time lags were similar; therefore, it is possible to evaluate the effect of time lag on both growth and circadian rhythm using PLA. Image measurement also has a lower cost than bioluminescence measurement. Therefore, the measurement of circadian rhythms using image measurement will make our proposed method more practical.

## Data availability statement

The original contributions presented in the study are included in the article/**Supplementary Material**. Further inquiries can be directed to the corresponding author.

## Author contributions

KM, TY, and HF designed this study. KM, TY, and YK performed experiments. KM and TY analyzed the data. KM and HF wrote the manuscript. All authors contributed to the article and approved the submitted version.

## Funding

This study was partially supported by Grants-in-Aid for JSPS Fellows (No. 18J20079 to KM) and Grants-in-Aid for Scientific Research (20H00423, 20H05424, 20H05540, 21H02317, and 22H04731 to HF) provided by Japan Society for the Promotion of Science.

## Acknowledgments

We are grateful to Prof. Norihito Nakamichi for creation of transgenic lines.

## Conflict of interest

The authors declare that the research was conducted in the absence of any commercial or financial relationships that could be construed as a potential conflict of interest.

## Publisher’s note

All claims expressed in this article are solely those of the authors and do not necessarily represent those of their affiliated organizations, or those of the publisher, the editors and the reviewers. Any product that may be evaluated in this article, or claim that may be made by its manufacturer, is not guaranteed or endorsed by the publisher.
