# Supplementary material for: Application of time lags between light and temperature cycles for growth control based on the circadian clock of Lactuca sativa L. seedlings
Source: Front Plant Sci. 2022 Oct 13;13:994555. doi: 10.3389/fpls.2022.994555 (PMC9802636; doi:10.3389/fpls.2022.994555)
Supplement: Supplementary file 1 [file DataSheet_1.zip › Pictures.pptx]

## Slide 1
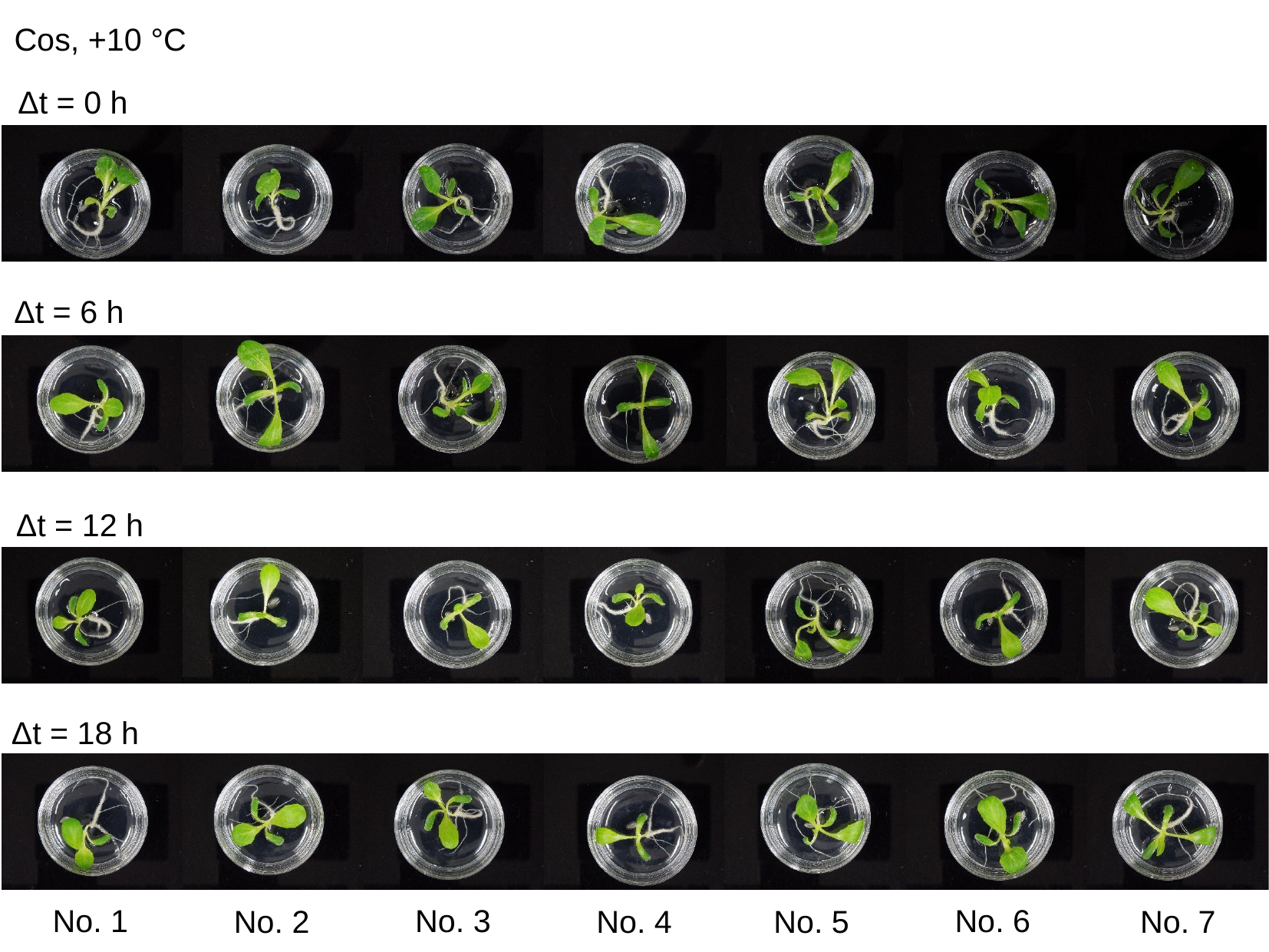

Cos, +10 °C
Δt = 0 h
Δt = 6 h
Δt = 12 h
Δt = 18 h
No. 3
No. 6
No. 1
No. 2
No. 4
No. 7
No. 5

## Slide 2
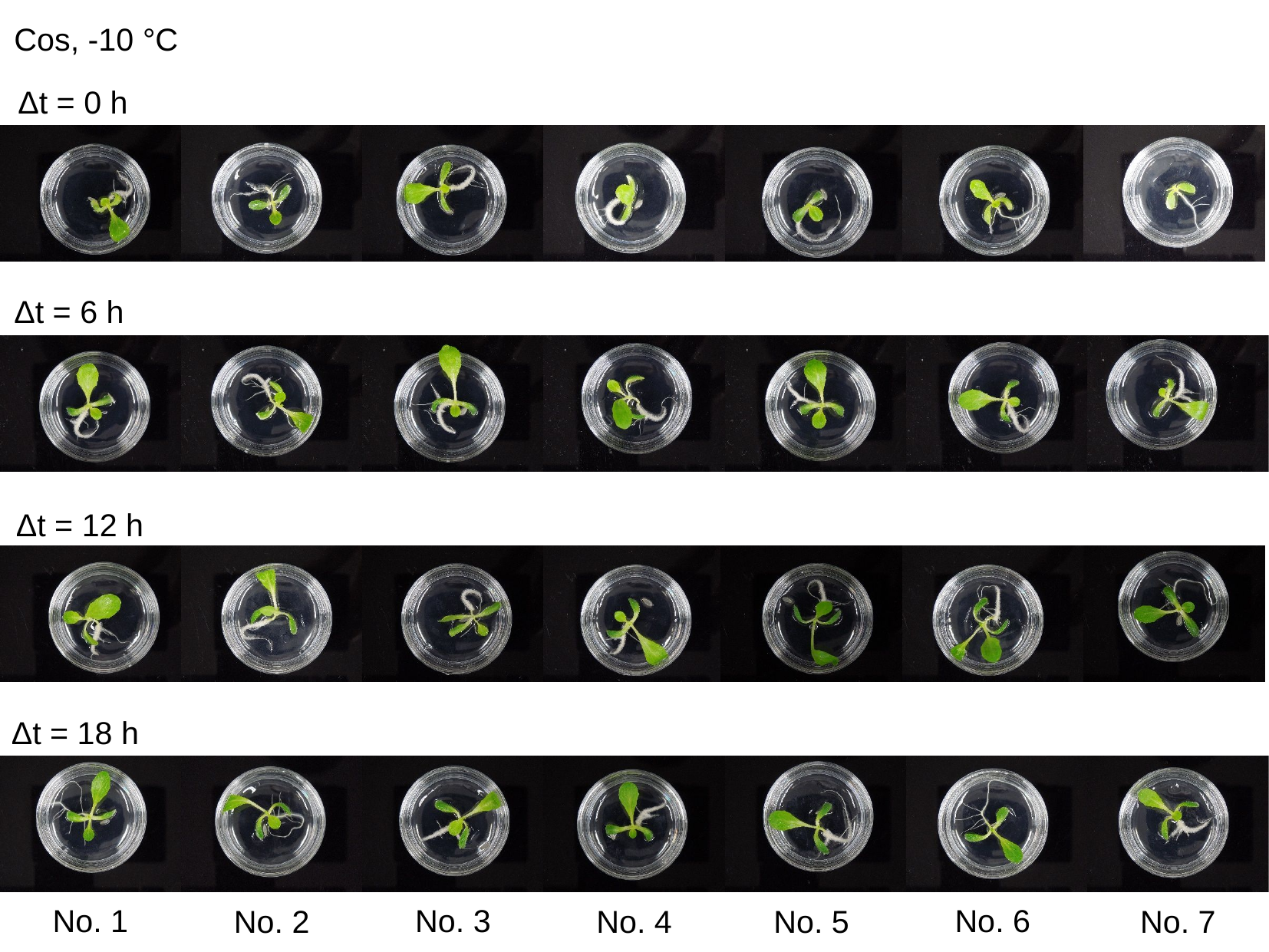

Cos, -10 °C
Δt = 0 h
Δt = 6 h
Δt = 12 h
Δt = 18 h
No. 3
No. 6
No. 1
No. 2
No. 4
No. 7
No. 5

## Slide 3
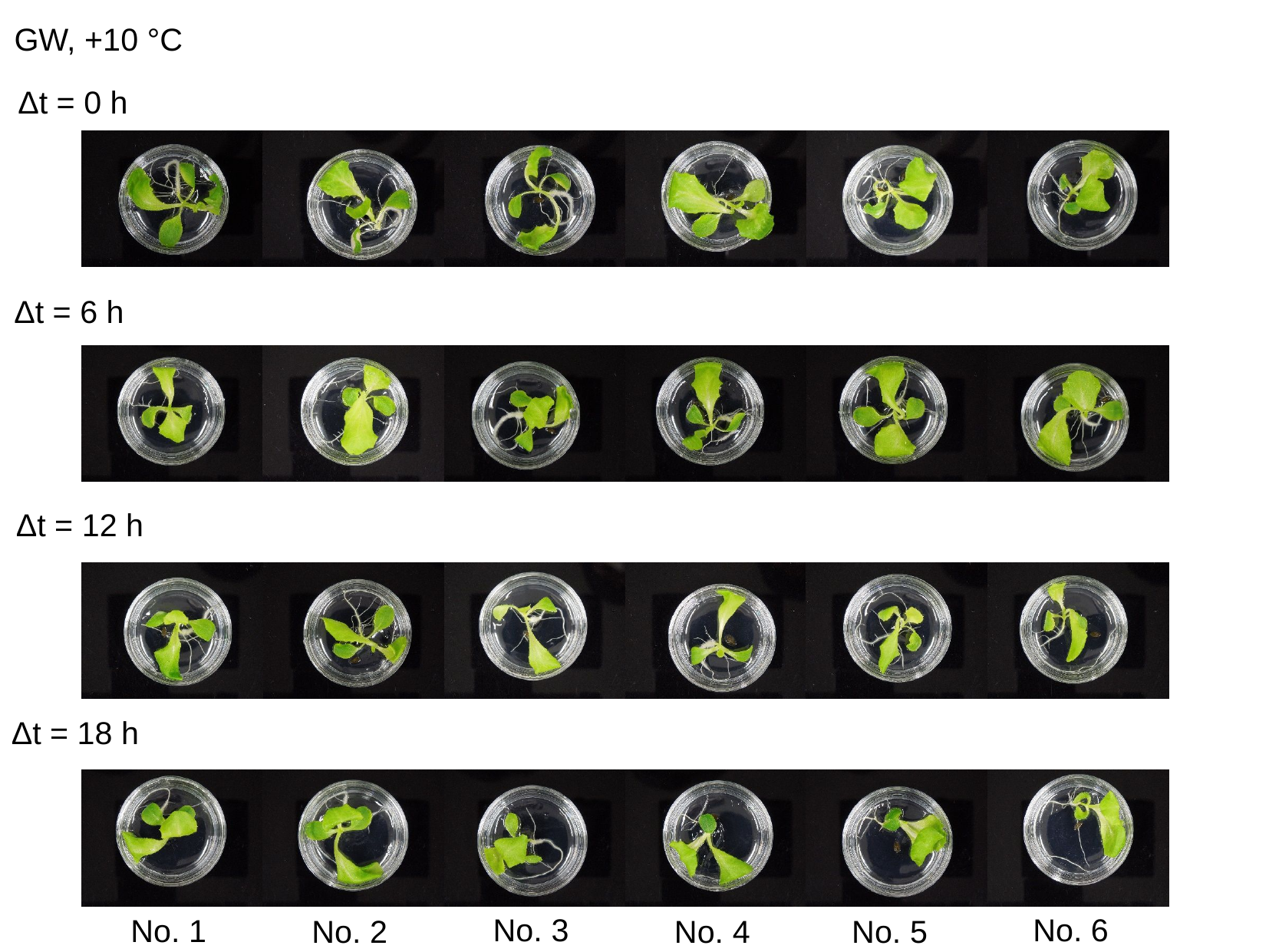

GW, +10 °C
Δt = 0 h
Δt = 6 h
Δt = 12 h
Δt = 18 h
No. 3
No. 6
No. 1
No. 2
No. 4
No. 5

## Slide 4
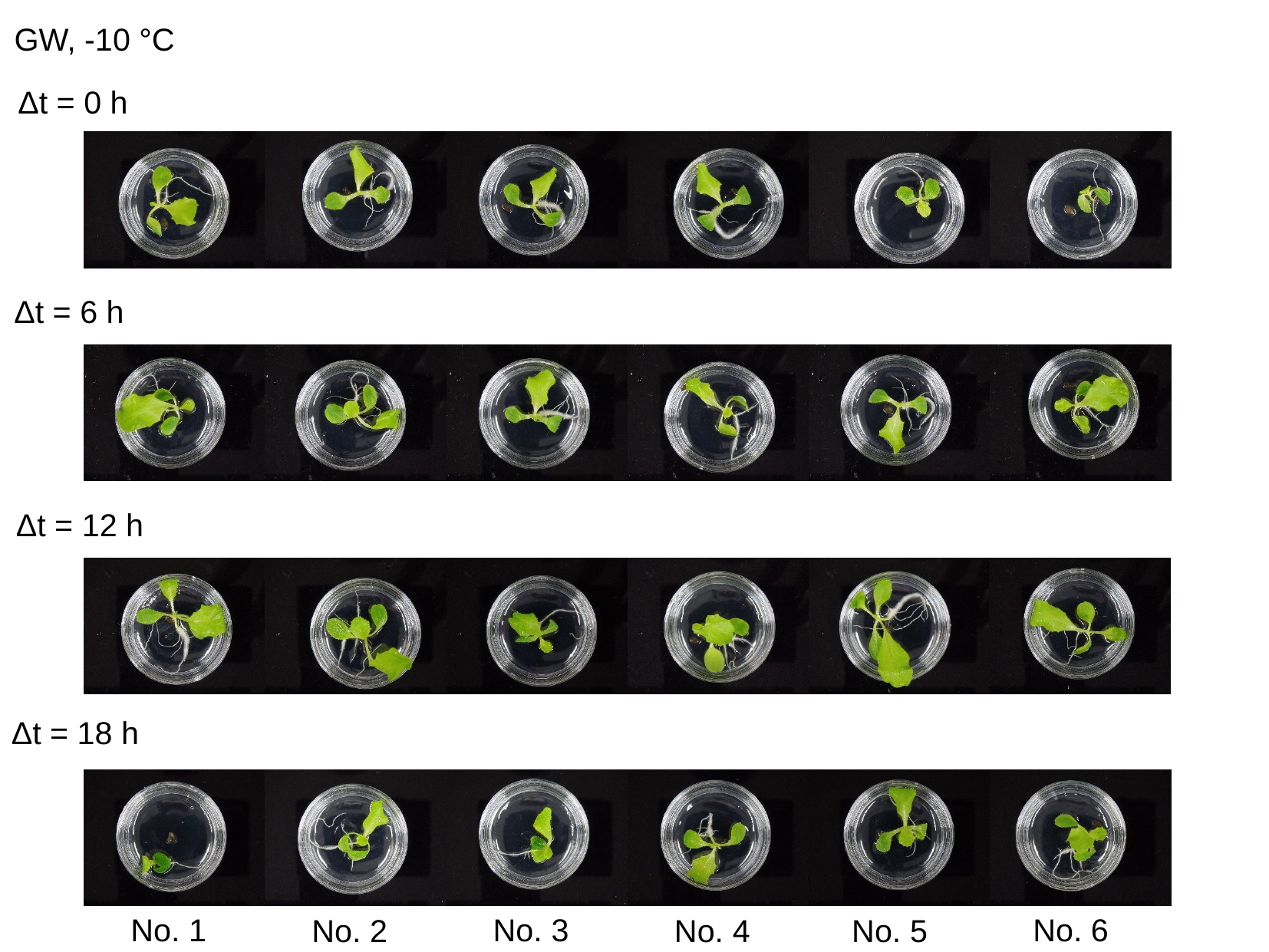

GW, -10 °C
Δt = 0 h
Δt = 6 h
Δt = 12 h
Δt = 18 h
No. 3
No. 6
No. 1
No. 2
No. 4
No. 5
