# Supplementary material for: Application of time lags between light and temperature cycles for growth control based on the circadian clock of Lactuca sativa L. seedlings
Source: Front Plant Sci. 2022 Oct 13;13:994555. doi: 10.3389/fpls.2022.994555 (PMC9802636; doi:10.3389/fpls.2022.994555)
Supplement: Supplementary file 2 [file Presentation_1.pdf]

Supplementary Material

1 Supplementary Figures

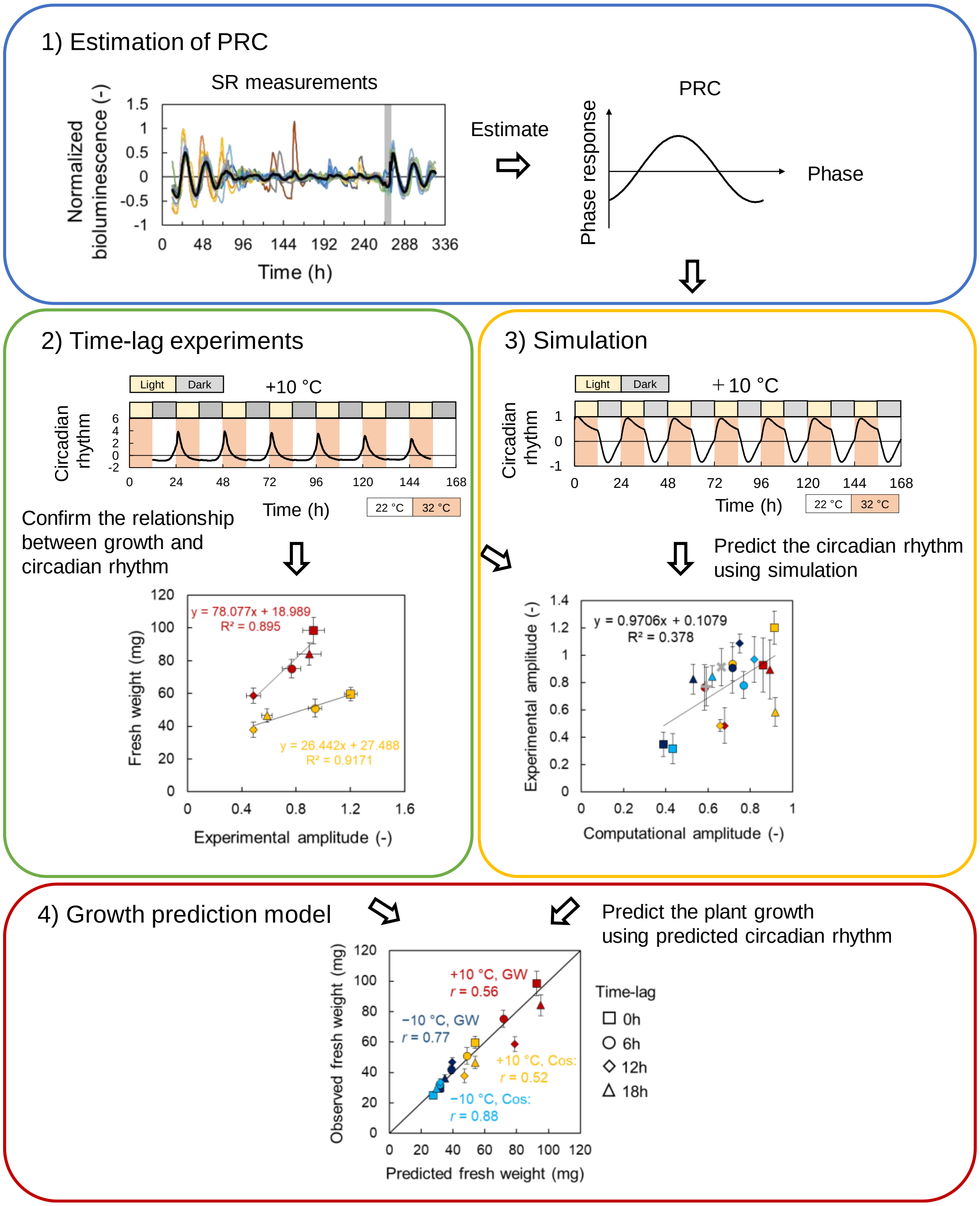

Figure S1. Overview of the construction of the growth prediction model for environmental time lags.

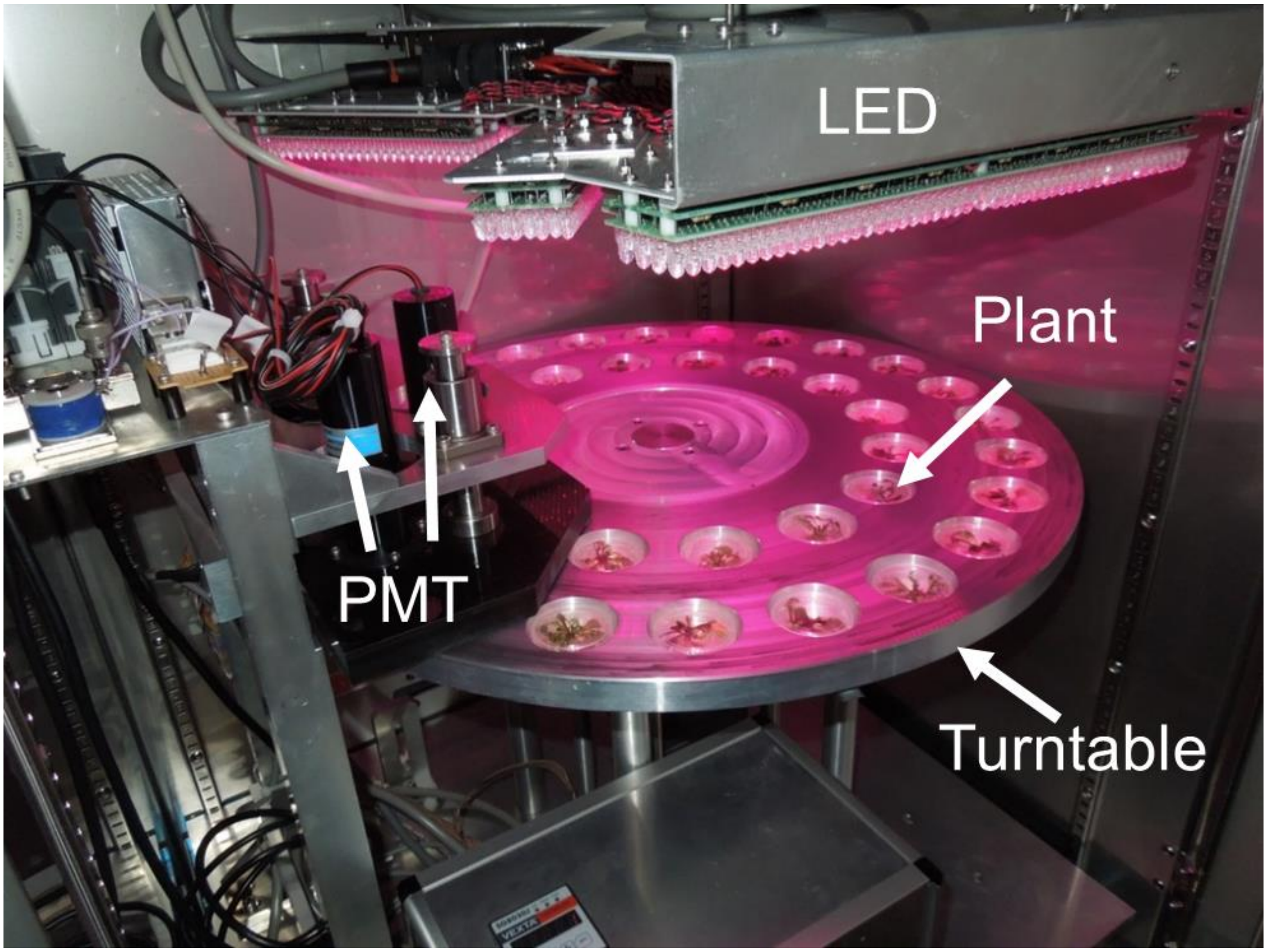

Figure S2. An automatic luminescence measuring system, *Kondotron*. Photomultiplier tubes (PMT) are enclosed in a light-tight box. Turntable is located under the PMT and rotates sequentially every 20 min under the control of a computer. Light-emitting diode (LED) illumination is also computer-controlled. *Kondotron* is installed in a temperature-controlled chamber. The plants in this picture are *Arabidopsis thaliana.*

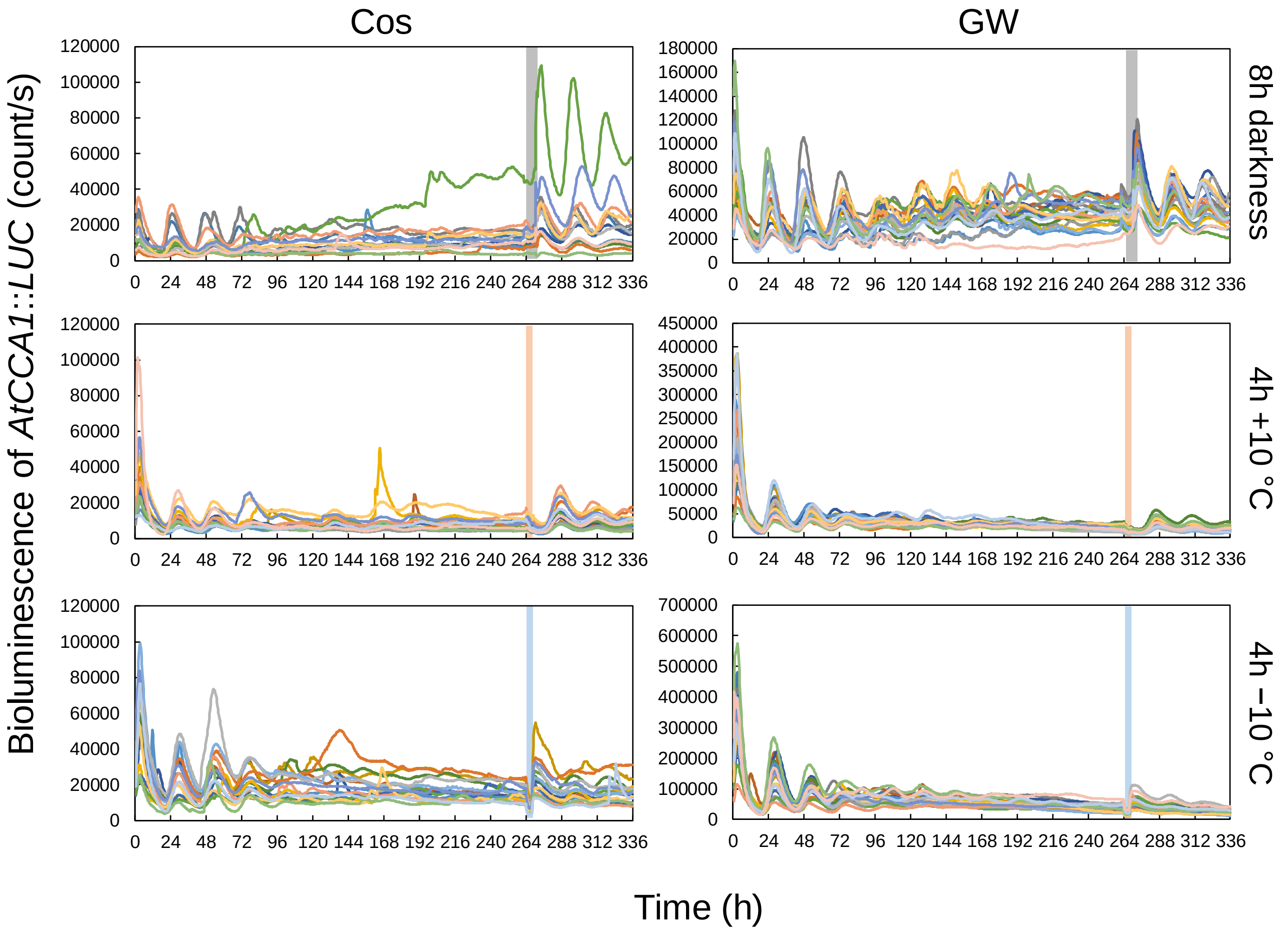

Figure S3. Bioluminescence in the singularity response in *Lactuca sativa* L. (cv. Cos and Greewave). Vertical bars represent the stimulation.

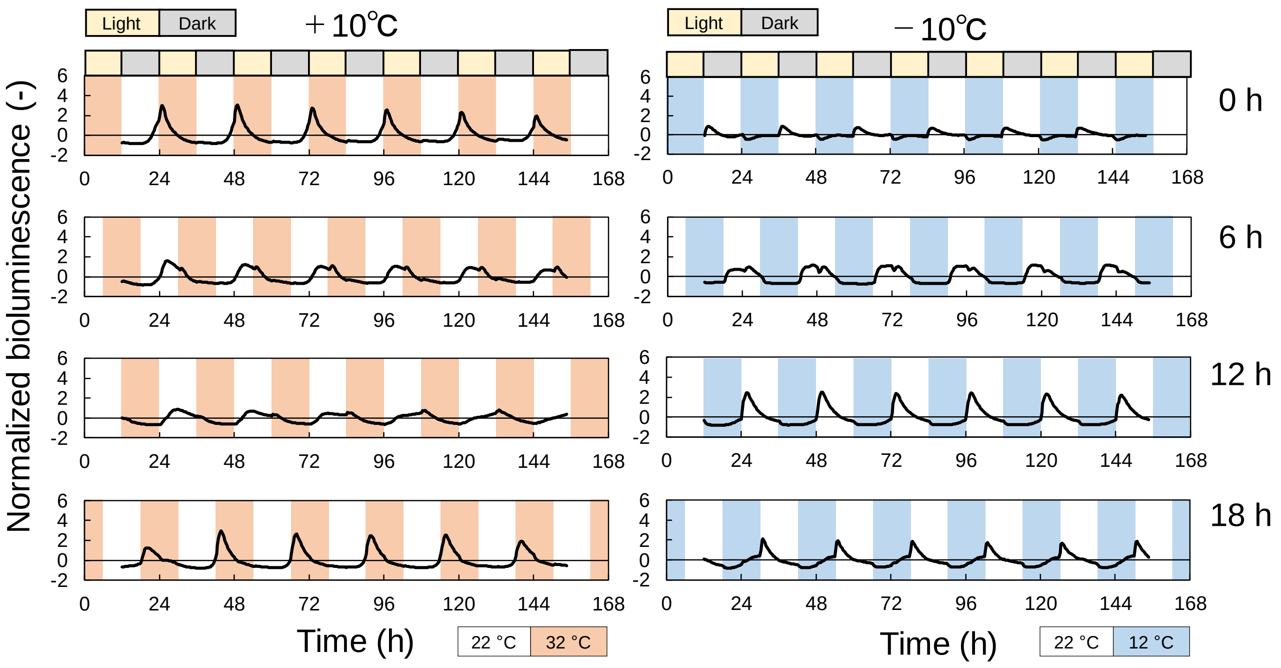

Figure S4. Normalized bioluminescence of *AtCCA1*::*LUC* in *Lactuca sativa* L. 'GW' under the light and ±10 °C temperature cycles with different time lags during an experiment to investigate the effect of time lags in light: dark and temperature cycles during cultivation. Each data is the average of 6 individuals.

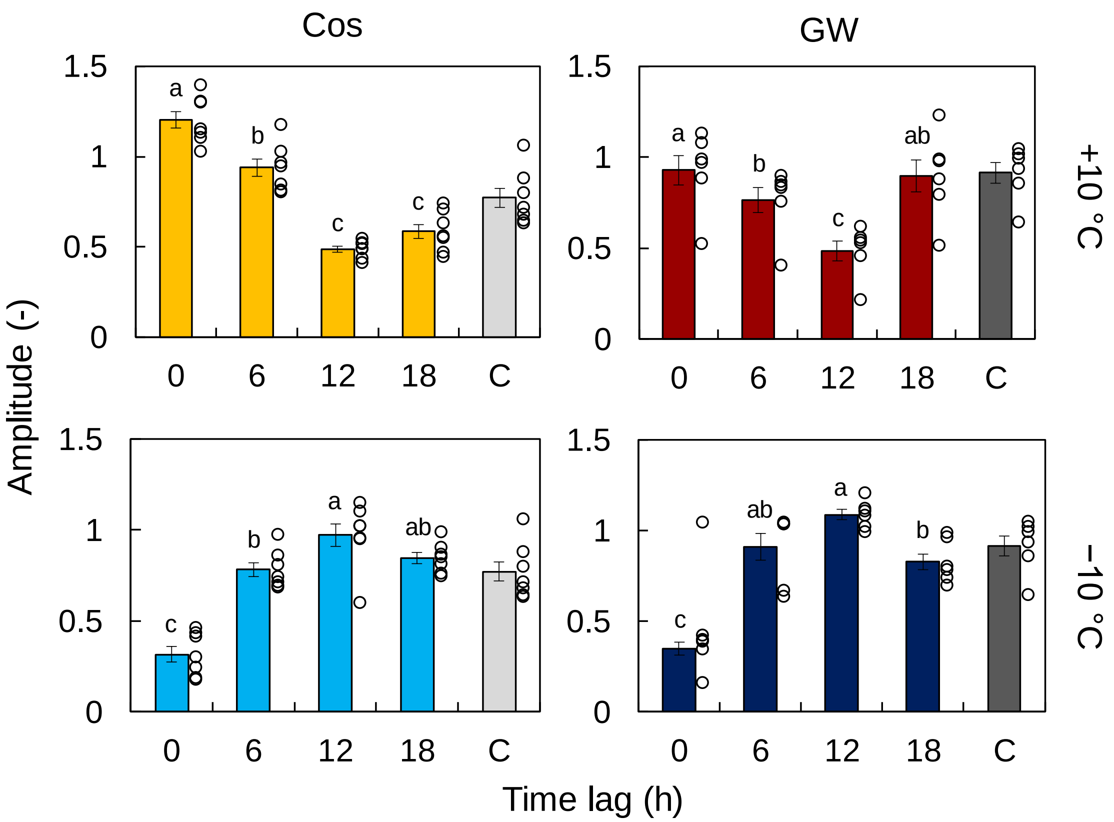

Figure S5. Effect of the time lag on amplitude of circadian rhythm. The control condition is labeled C. Each data is mean of n = 7 individuals in *Lactuca sativa* L. 'Cos' and 6 in 'GW'. Error bars indicate standard error. The circles indicate the individual data points. Two conditions that do not have the same letter indicate significant differences for each panel (Tukey-Kramer test, $p < 0.05$).

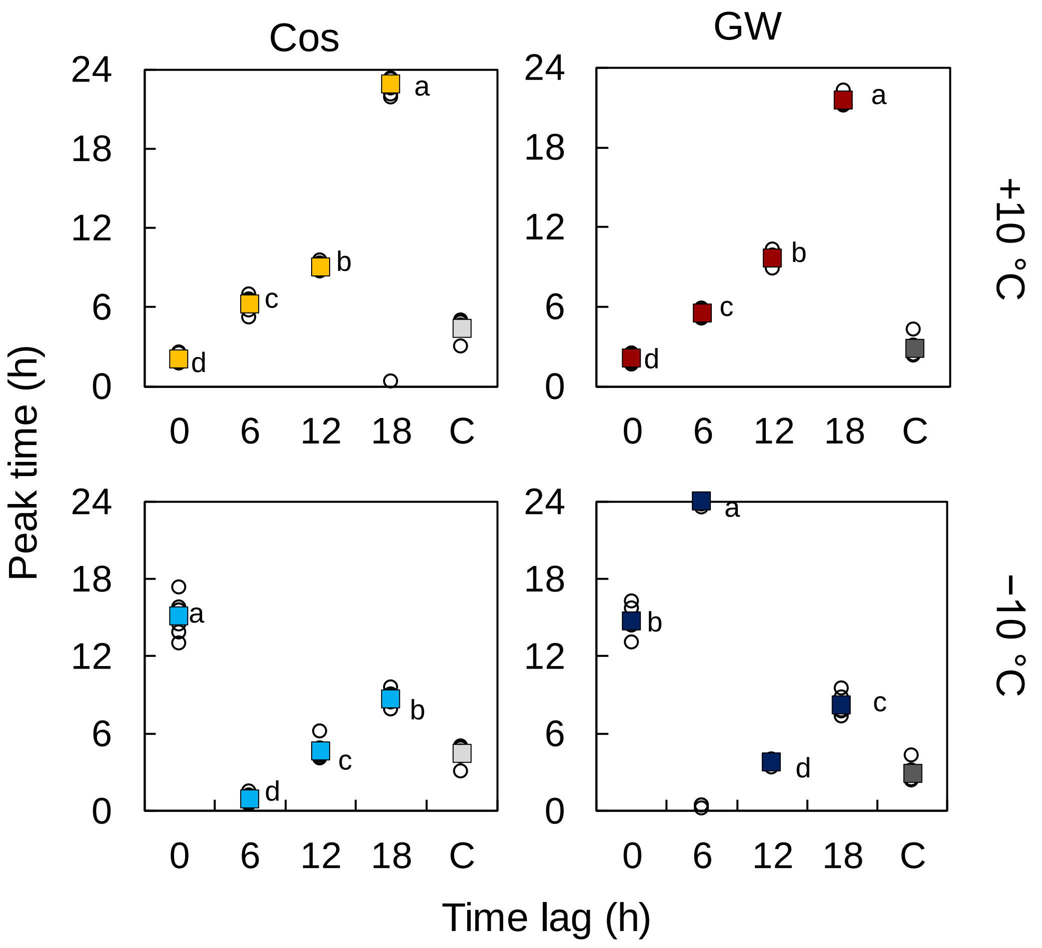

Figure S6. Effect of the time lag on phase of circadian rhythm. The control condition is labeled C. Each data is circular mean of $n = 7$ individuals in *Lactuca sativa* L. ‘Cos’ and 6 in ‘GW’. The circles indicate the individual data points. Two conditions that do not have the same letter indicate significant differences for each panel (Watson-Williams test with Bonferroni correction, $p < 0.05$).

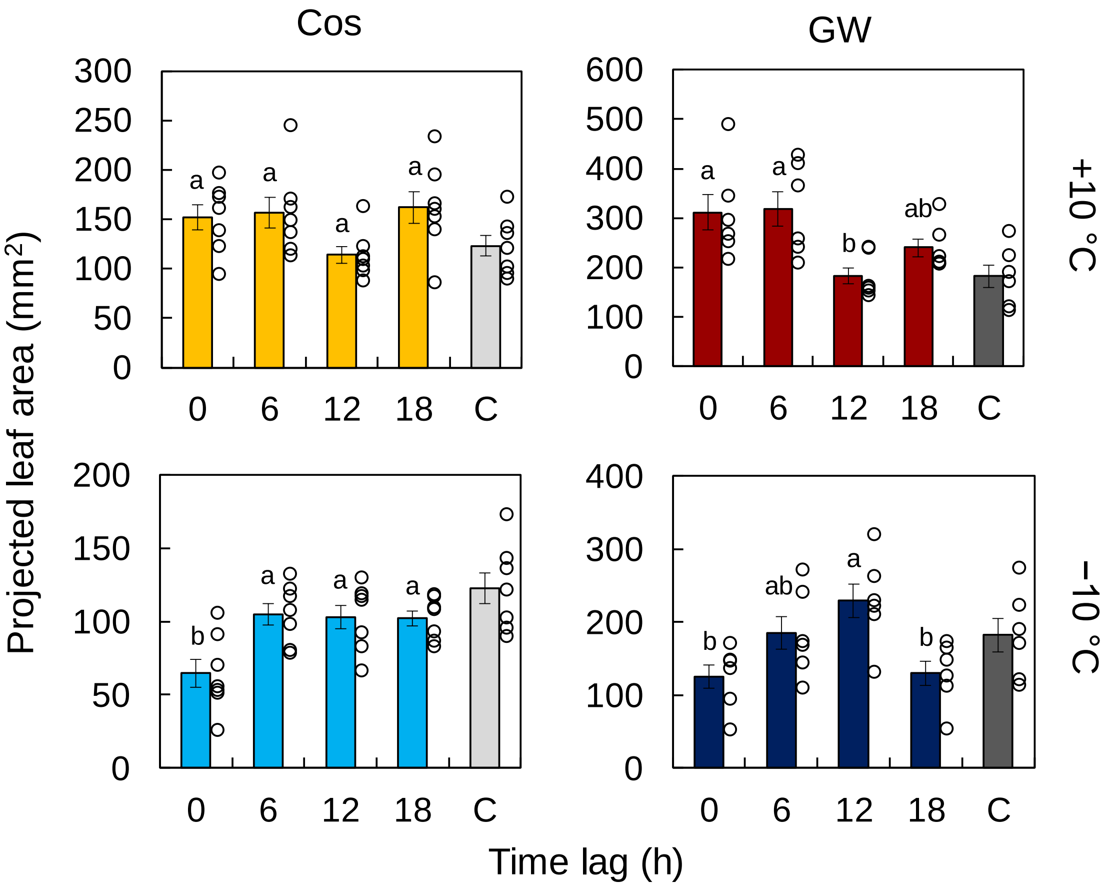

Figure S7. Effect of the time lag on projected leaf area (PLA). The control condition is labeled C. Each data is mean of $n = 7$ individuals in *Lactuca sativa* L. 'Cos' and 6 in 'GW'. Error bars indicate standard error. The circles indicate the individual data points. Two conditions that do not have the same letter indicate significant differences for each panel (Tukey-Kramer test, $p < 0.05$).

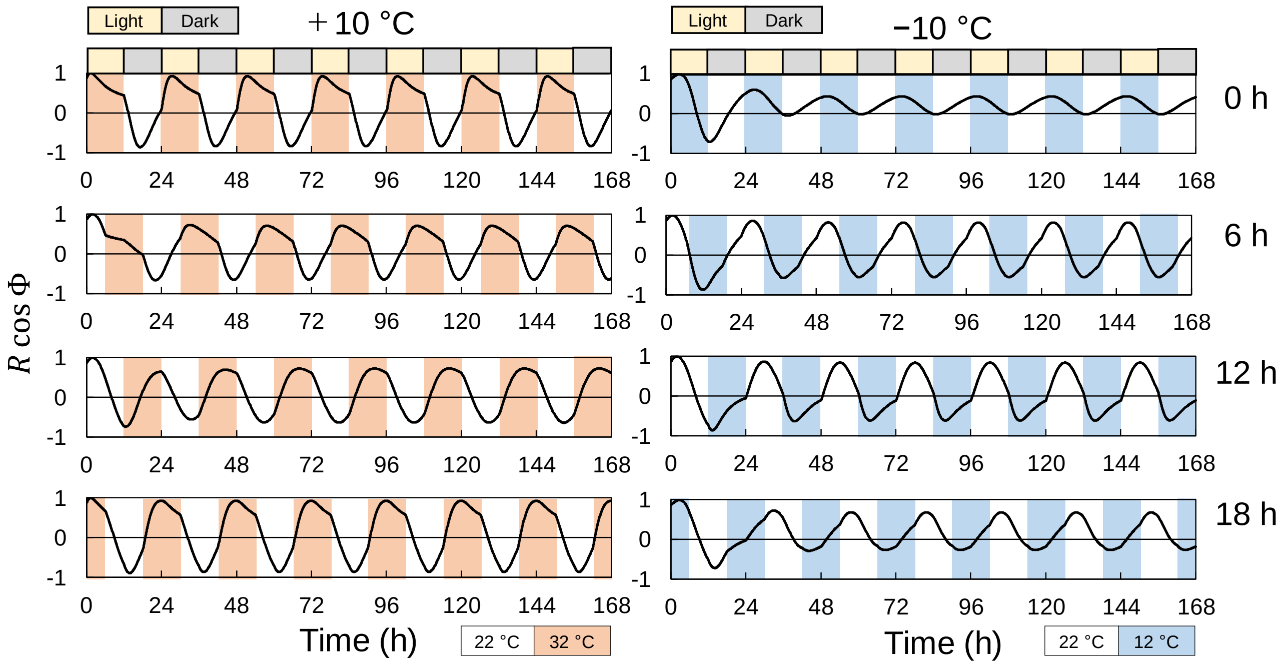

Figure S8. Simulation of *AtCCA1* rhythm using the parameters of circadian rhythm in *Lactuca sativa* L. 'Cos' under the light and ±10 °C temperature cycles with different time lags. The parameters used in this simulation are listed in Tables S1 and S2.

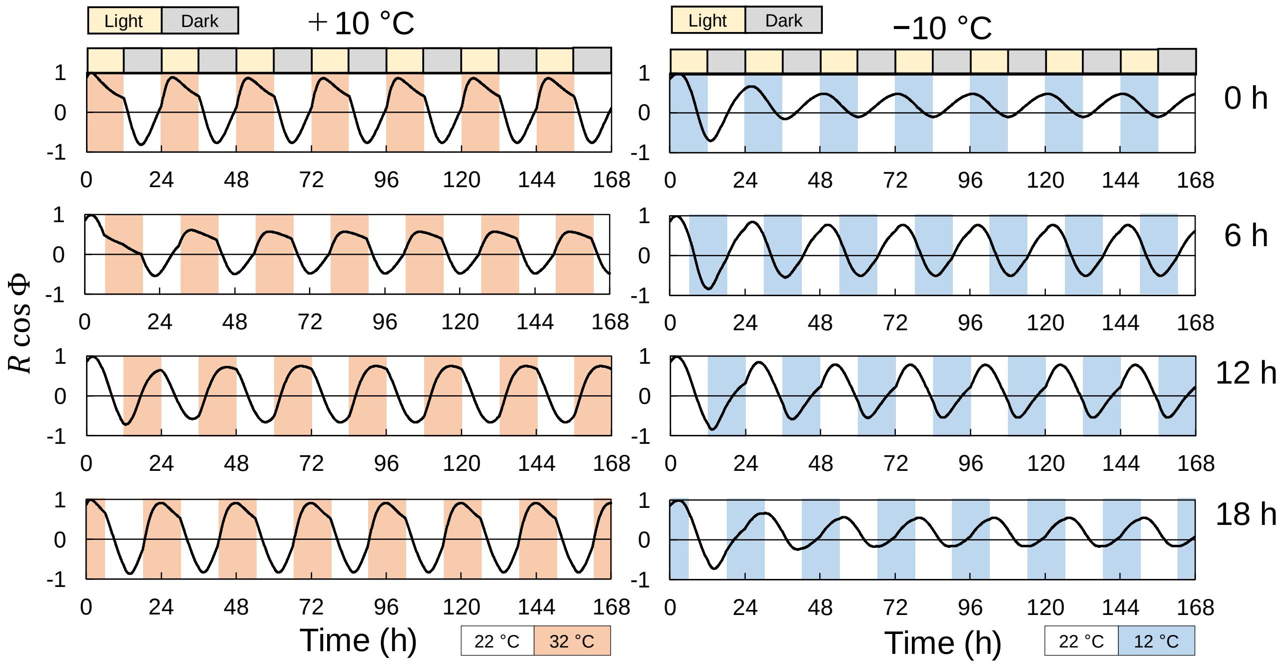

Figure S9. Simulation of *AtCCA1* rhythm using the parameters of circadian rhythm in *Lactuca sativa* L. 'GW' under the light and ±10 °C temperature cycles with different time lags. The parameters used in this simulation are listed in Tables S1 and S2.

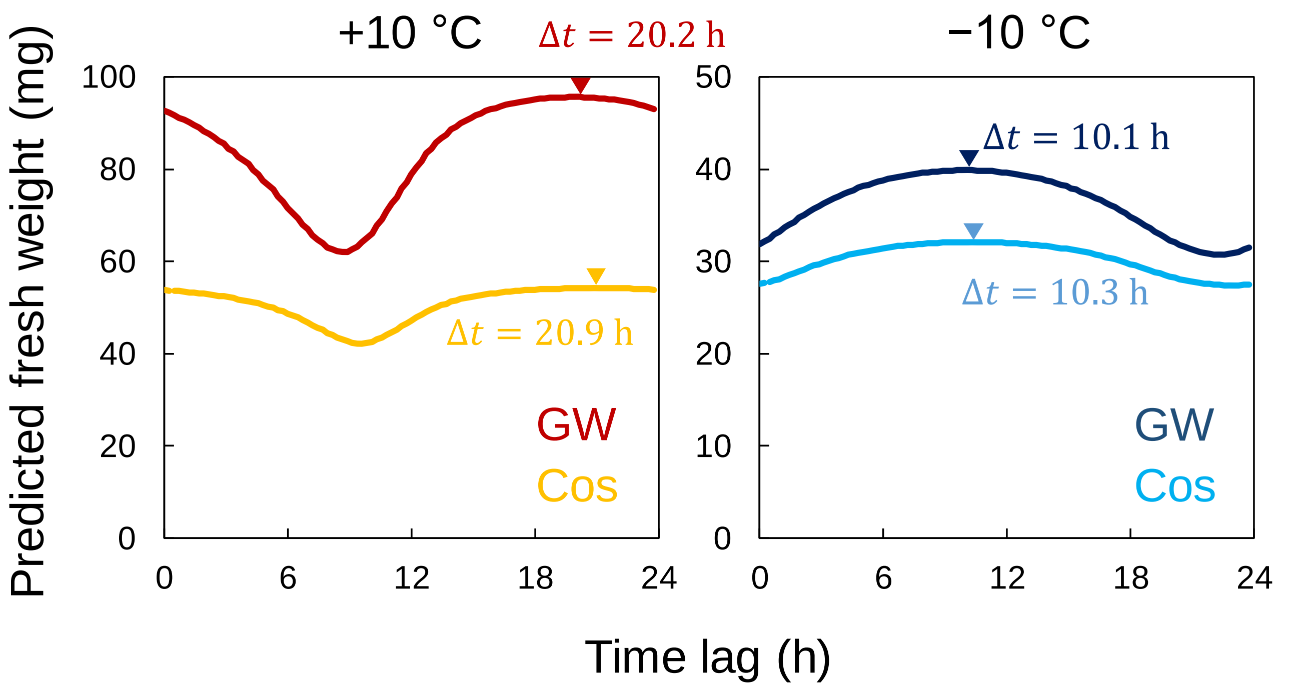

Figure S10. Predicted fresh weight based on the simulation using PRC. Triangles indicate the time lags where predicted fresh weight is the highest.

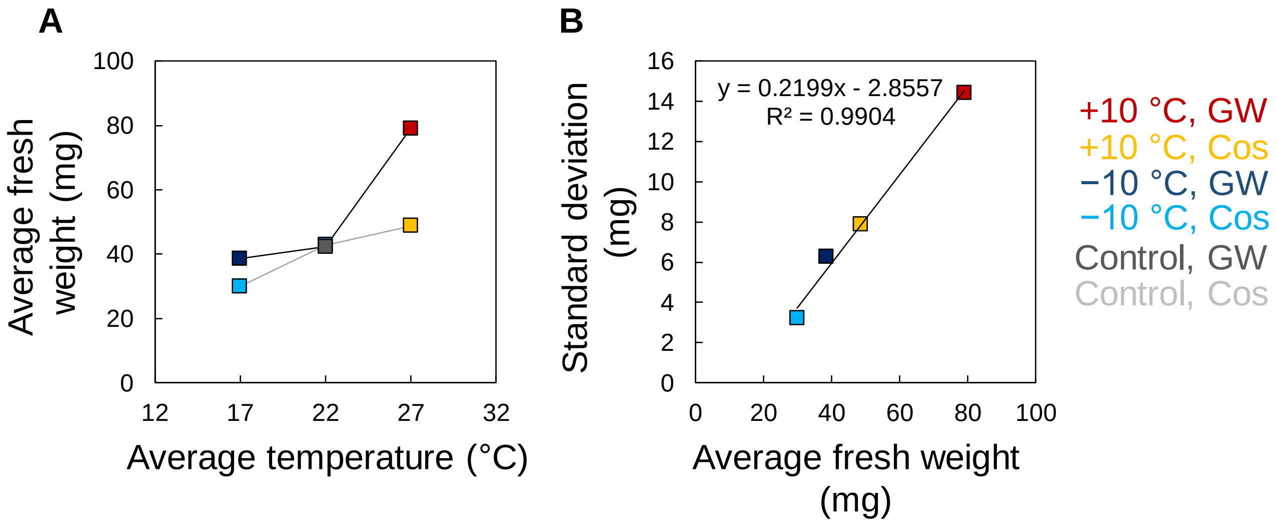

Figure S11. Average fresh weight and standard deviation in each time-lag experiments. **(A)** Average fresh weight under the different average temperature. Gray and black lines indicate the changes in fresh weight of *Lactuca sativa* L. 'Cos' and 'GW', respectively. The average fresh weights at average temperature 17 and 27 °C represent the average value of the results at four time-lag (Δt = 0, 6, 12, 18 h) in the −10 °C and +10 °C conditions. The fresh weight at average temperature 22 °C represents that in the control condition. **(B)** The standard deviation of the fresh weights at four time-lag (Δt = 0, 6, 12, 18 h). The black line represents regression line and R^2 is the coefficient of determination.

2 Supplementary Tables

Table S1. Period and amplitude of circadian rhythm in *Lactuca stave* L. and *Arabidopsis thaliana*.

Species (Varieties)	Period ± SD (h)	Amplitude ± SD	*n*
Lactuca sativa L. (Cos)	23.83 ± 1.35	0.542 ± 0.158	31
Lactuca sativa L. (GW)	24.33 ± 1.62	0.693 ± 0.124	40
A. thaliana	22.77 ± 0.35	1.023 ± 0.083	27

Table S2. SR parameters to the 8-h darkness and 4-h ± 10 °C stimulation. Two conditions that do not have the same letter indicate significant differences for stimuli (Tukey-Kramer test for *R'* and Watson-Williams test with Bonferroni correction for Θ', $p < 0.05$).

Species (Varieties)	8 h darkness		4 h + 10 ℃		4 h + 10 ℃	
	R'	Θ' (CT)	R'	Θ' (CT)	R'	Θ' (CT)
Lactuca sativa L. (Cos)	0.334 a	21.0 ab	0.334 a	4.7 a	0.171 a	17.5 a
Lactuca sativa L. (GW)	0.276 a	22.2 b	0.296 a	4.7 a	0.120 b	18.1 a
A. thaliana	0.358 a	20.7 a	0.392 b	5.7 b	0.122 b	19.2 b

Table S3. Estimated PRC parameters to the 8-h darkness and 4-h ± 10 °C stimulation.

Species (Varieties)	8 h darkness		4 h + 10 ℃		4 h + 10 ℃	
	a	α (rad/2π)	a	α (rad/2π)	a	α (rad/2π)
Lactuca sativa L. (Cos)	0.179	0.138	0.314	0.534	0.148	0.060
Lactuca sativa L. (GW)	0.144	0.182	0.271	0.532	0.103	0.086
A. thaliana	0.195	0.128	0.388	0.579	0.105	0.133
